# New faunal data on lacewings (Insecta, Neuroptera) collected from Saudi Arabia

**DOI:** 10.3897/zookeys.936.49962

**Published:** 2020-05-28

**Authors:** Agostino Letardi, Mahmoud S. Abdel-Dayem, Hathal M. Al Dhafer

**Affiliations:** 1 ENEA C.R. Casaccia, Roma, 00123, Italy ENEA C.R. Casaccia Roma Italy; 2 College of Food and Agricultural Sciences, King Saud University, Riyadh, 11451, Saudi Arabia King Saud University Riyadh Saudi Arabia; 3 Entomology Department, Faculty of Science, Cairo University, Giza, 12613, Egypt Cairo University Giza Egypt

**Keywords:** distribution, endemic, new records, Neuroptera, Saudi Arabia

## Abstract

This study presents new data on the lacewing fauna of Saudi Arabia based on field work performed between 2014 and 2019. Sixty-one lacewing species from 37 genera and seven Neuroptera families were documented. Additionally, two species belonging to *Dielocroce* and *Pseudomallada* were identified only to genus level. Three of the identified species are new records to Saudi Arabia (*Aspoeckiella gallagheri* Hölzel, 2004, *Bankisus maculosus* Hölzel, 1983, and *Nemoleon secundus* Hölzel, 2002). Another three species are new to the fauna of the Arabian Peninsula (*Mantispa aphavexelte* Aspöck & Aspöck, 1994, *Omoleon jeanneli* Navás, 1936, and *Stylascalaphus krueperi* van der Weele, 1909). The first reports of eight species are provided after their original description from Saudi Arabia; namely, *Creoleon ultimus* Hölzel, 983, *Cueta amseli* Hölzel, 1982, *Cu. asirica* Hölzel, 1982, *Distoleon asiricus* Hölzel, 1983, *Geyria pallida* Hölzel, 1983, *Neuroleon delicatus* Hölzel, 1983, *N. virgineus* Hölzel, 1983 and *Solter buettikeri* Hölzel, 1982 Zoogeographically, most lacewing species documented in the Arabian Peninsula are endemic (26.2%), followed by Afro-syroeremic (23.0%), Afrotropical (18.0%), and Afro-syro-iranoeremic (14.8%) species. Palaearctic species (4.9%) had the lowest contribution.

## Introduction

**Figure 1.**
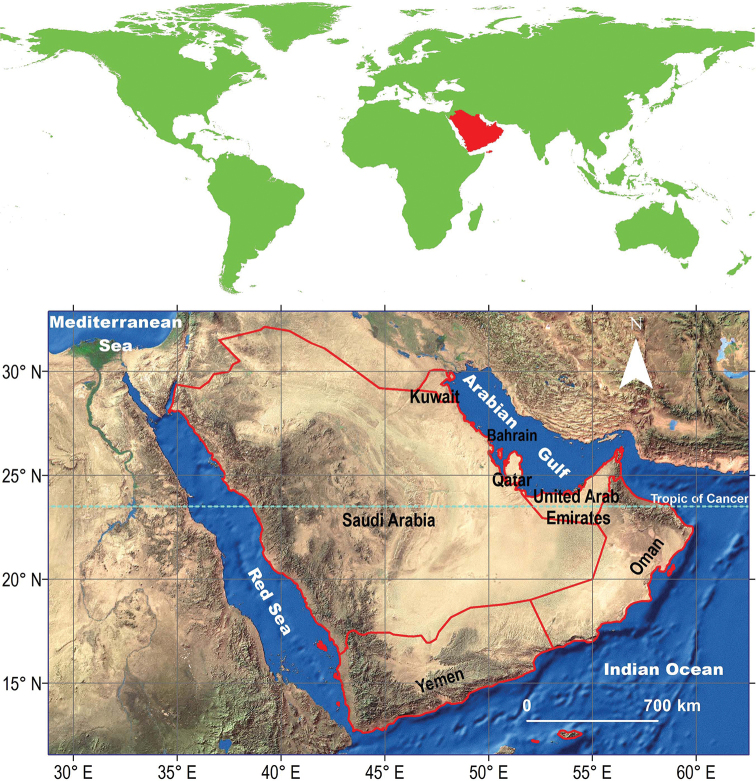
Map of the Arabian Peninsula.

The Arabian Peninsula is located on the Arabian tectonic plate in northeast Africa, western Asia. Its fauna has different zoogeographical affinities (Larsen 1984; Hölzel 1998), as it lies at the convergence of three zoogeographical realms: the Palaearctic from the north, the Afrotropical from the southwest, and the Oriental from the east. The Arabian Peninsula covers a surface area of 3.2 million km^2^, and encompasses Bahrain, Kuwait, Oman, Qatar, Saudi Arabia, United Arab Emirates, and Yemen (Fig. 1). Saudi Arabia covers approximately two thirds (1,969,000 km^2^) of the peninsula and is considered to hold the richest biodiversity in it (Miller 1994; Mallon 2011). The key biological sites in Saudi Arabia include isolated mountain massifs, rawdahs (meadows), wadis (valleys), juniper woodlands, acacia woodlands, freshwater wetlands, salt marshes, mangrove thickets, marine islands, coral reefs, algal beds, and sea grass beds (Abuzinada et al. 2005).

Neuroptera is a small group of insects that currently contains ca. 5,800 species in 19 families (Oswald 2019). Nine families have been reported to occur in the Arabian Peninsula: Chrysopidae, Hemerobiidae, Sisyridae, Coniopterygidae, Mantispidae, Berothidae, Nemopteridae, Myrmeleontidae, and Ascalaphidae (the last two recently fused in a single family, see Machado et al. 2018). Saudi Arabia has an exceptionally rich fauna of aridophilic families, Nemopteridae and Myrmeleontidae, with studies over the last 40 years documenting several new species (Meinander 1980; Hölzel 1982, 1983a, 1988, 1998, 1999, 2001, 2004). For many of these species, the original description represents the only published data. Consequently, despite many studies documenting Neuroptera in the Arabian Peninsula over the last 40 years (Meinander 1979; Hölzel 1980, 1983b; Sziráki 1992, 1997; Aspöck and Aspöck 1998; Sziráki and van Harten 2006; Saji and Whittington 2008), there is still a paucity of faunal surveys focusing on Neuroptera that could provide important distribution and ecological information on this group.

Over the last six years, new faunal unpublished records of lacewings have been gathered in Saudi Arabia. Thus, this study aims to provide new information on this group in Saudi Arabia, with a focus on poorly known and rarely collected species.

## Materials and methods

The collection of samples was conducted between 2014 and 2019 at different locations in Saudi Arabia. Adult lacewings were captured mainly with light traps (LT), but also pitfall traps (PT), sugar traps (ST), and sweep nets (SW) were utilized. Specimens were preserved by desiccation or in 70% alcohol. They were then deposited in the collections of King Saud University Museum of Arthropods (**KSMA**) (Riyadh, Saudi Arabia) unless otherwise indicated (AL: Agostino Letardi collection). The species are presented in systematic order, by family and genus, according to Aspöck et al. (2001).

For the examined material, the following information were verified: Saudi Province (in bold) followed by a colon (:), the name of the governorate, locality, geographical coordinates (latitude, longitude), elevation (m), date of collection, capture technique(s), collector(s), number of examined specimens followed by sex (if determined) or “ex” (if the specimen sex could not be recognized because the abdomen lost or other reasons). The examined material was arranged by province, governorate, and locality name, in alphabetical order. Then, it was presented in ascending order according to altitude, and chronologically based on month of collection. When the records were from different provinces and governorates, a full stop separate them. A semicolon was used to separate different records. The governorate name was only cited at the beginning if the records were from the same governorate. Labels that had the same locality name, except for slight differences (such as elevation, collection date, collector/s), were listed jointly with the second label, specified with “*ibidem*”, and followed by a comma and the different data. The global distribution was derived from Oswald (2019) and general range was sourced from Aspöck et al. (2001). A biology entry summarizes previous knowledge on habitat, host, behavior, etc., while a notes entry provides novel information on distribution, habitat, taxonomy, and other relevant data.

## Results

### Chrysopidae Schneider, 1851

#### Italochrysa bimaculata

Taxon classificationAnimaliaNeuropteraChrysopidae

Hölzel, 1980

5E3A744D-2D32-5390-B98D-5609F9E041E7

##### Material examined.

**Al Bahah Province**: Al Mekhwah, Shada Al-A’Ala Nature Reserve, 19°52.598'N, 41°18.672'E, 892 m, 15 Oct 2015, LT, Al Dhafer et al. leg., 1♀; *ibidem*, 19°51.762'N, 41°18.089'E, 1225 m, 17 Oct 2014, LT, Al Dhafer et al. leg., 1♂; *ibidem*, 2 Nov 2015, LT, Al Dhafer et al. leg., 1♀; *ibidem*, 19°50.710'N, 41°18.267'E, 1474 m, 3 Nov 2013, LT, Al Dhafer et al. leg., 1♂; *ibidem*, 14 Nov 2015, LT, Al Dhafer et al. leg., 2♂; *ibidem*, 19°50.411'N, 41°18.686'E, 1611 m, 17 Nov 2014, LT, Al Dhafer et al. leg., 1♂; *ibidem*, 19°50.575'N, 41°18.691'E, 1666 m, 3 Apr 2014, LT, Al Dhafer et al. leg., 1♀; *ibidem*, 5 May 2015, LT, Al Dhafer et al. leg., 1♀; Wadi Reyam (NE Al Makhwah), 19°50'28"N, 41°22'34"E, 473 m, 7 Apr 2019, LT, D. Baiocchi et al. leg., 1♀. **Asir Province**: Abha, Garf Raydah Nature Reserve, 18°11.749'N, 42°23.345'E, 1614 m, 5 Sep 2015, LT, Al Dhafer et al. leg., 1♂; *ibidem*, 18 Nov 2015, LT, Al Dhafer et al. leg., 1♂; Wadi Marabah (WSW Abha), 18°10.293'N, 42°22.195'E, 1150 m, 4 Apr 2017, LT, M.S. Abdel-Dayem leg., 1♂; *ibidem*, 18°10.18'N, 42°22.12'E, 1197 m, 1 Apr 2017, LT, D. Baiocchi leg., 2♂; *ibidem*, 11‒13 Apr 2019, LT, D. Baiocchi et al. leg., 1♀ (AL). Khamis Mushait, Wadi Ibn Hashbal (14 km N Khamis Mushait), 18°27.34'N, 42°42.53'E, 1926 m, 2 Apr 2017, LT, D. Baiocchi leg., 1♂.

##### Distribution.

Africa: Senegal, Tunisia. Asia: Israel, Saudi Arabia, Yemen. A polycentric Afrotropical species.

##### Notes.

This species was previously reported as *I. arabica* in Al Bahah Province (Hölzel 1980). The listed specimens were collected between 1,150 and 1,926 m elevation and seem to be associated with *Acacia* woodlands and rocky areas with Barbary fig shrubs (*Opuntia ficus-indica* (L.) Mill. (Cactaceae)) in the mountainous areas of southwest of Saudi Arabia.

#### Pseudomallada amseli

Taxon classificationAnimaliaNeuropteraChrysopidae

(Hölzel, 1980)

75375C9F-5A0C-5B38-897E-CC76FB5EEBA9

##### Material examined.

**Asir Province**: Abha, Wadi Marabah (WSW Abha), 18°10.18'N, 42°22.12'E, 1197 m, 1 Apr 2017, LT, D. Baiocchi leg., 4♀; *ibidem*, 1♀ (AL).

##### Distribution.

Africa: Ethiopia. Asia: Israel, Oman, Saudi Arabia, Yemen. A possible Afrotropical species.

##### Notes.

It was previously reported in the provinces of Al Bahah and Asir (Hölzel 1980). The listed specimens were collected at 1197 m elevation in mountainous *Acacia* woodland areas in southwest Saudi Arabia.

#### Pseudomallada arabicus

Taxon classificationAnimaliaNeuropteraChrysopidae

(Hölzel, 1995)

4A11ADCB-F697-5406-9B93-5ED5FBEB8E73

##### Material examined.

**Al Bahah Province**: Al Mandaq, Wadi Tourabah (E An Na’Amah), 20°11'01"N, 41°18'42"E, 1826 m, 6 Apr 2019, LT, D. Baiocchi et al. leg., 4♀ and 3♂. **Asir Province**: Abha, Wadi Marabah (WSW Abha), 18°10.18'N, 42°22.12'E, 1197 m, 1 Apr 2017, LT, D. Baiocchi leg., 8♀; *ibidem*, 18°10.293'N, 42°22.195'E, 1150 m, 1 Apr 2017, LT, M.S. Abdel-Dayem leg., 9♀ and 2♂; *ibidem*, 1♀ and 1♂ (AL).

##### Distribution.

Asia: Saudi Arabia, Yemen. A possible Arabian endemic species.

##### Notes.

*Pseudomallada arabicus* was originally described from Fayfa Mountain in Jizan Province (Hölzel 1995). The listed specimens were collected in *Acacia* woodlands in the highlands (1150–1826 m elevation) of southwestern Saudi Arabia.

#### Pseudomallada spadix

Taxon classificationAnimaliaNeuropteraChrysopidae

(Hölzel, 1988)

3574C734-7455-564B-89D3-B0F89F4C1EE8

##### Material examined.

**Al Bahah Province**: Al Mandaq, Wadi Tourabah (E An Na’Amah), 20°11'01"N, 41°18'42"E, 1826 m, 6 Apr 2019, LT, D. Baiocchi et al. leg., 5♀ and 2♂. Al Mekhwah, Shada Al-A’Ala Nature Reserve, 19°51.682'N, 41°18.263'E, 1291 m, 29 Mar 2017, LT, M.S. Abdel-Dayem leg., 1♀; *ibidem*, 1♀ (AL). **Jizan Province**: Al Darb, Wadi Reem, 17°52.551'N, 42°16.664'E, 136 m, 5 Apr 2017, LT, M.S. Abdel-Dayem leg., 1♂.

##### Distribution.

Africa: Sudan. Asia: Oman, Saudi Arabia, Yemen. A possible Afrotropical species.

##### Notes.

It was originally described from the provinces of Al Bahah and Asir (Hölzel 1988). The listed specimens were collected in *Acacia* woodlands at low and mid elevations (136‒1826 m) in southwestern of Saudi Arabia.

#### Pseudomallada venosus

Taxon classificationAnimaliaNeuropteraChrysopidae

(Rambur, 1838)

D92AE2F7-DD05-5285-BAD8-2A7A1DAD9877

##### Material examined.

**Asir Province**: Rijal Almaa, Wadi Kasan (3 km N Al Hubail), 18°06.981'N, 42°13.939'E, 451 m, 3 Apr 2017, LT, M.S. Abdel-Dayem and I. Rasool leg., 2♀ and 1♂.

##### Distribution.

Africa: Algeria, Egypt, Morocco, Sudan, Tunisia. Asia: Afghanistan, Iran, Israel, Lebanon, Mongolia, Oman, Pakistan, Saudi Arabia, Turkey, Yemen. Europe: France, Portugal, Spain. It is a Palaearctic species.

##### Biology.

This green lacewing is generally associated with low vegetation in extremely dry-warm biotopes, predominantly in steppes and semidesert-like habitats, and is quite common at light traps (Aspöck et al. 1980; Diaz-Aranda and Monserrat 1990).

##### Notes.

This species was previously reported in several Saudi provinces: Asir, Al Bahah, Eastern Province, Madinah, Makkah, and Riyadh (Hölzel 1988). The listed adult specimens were collected by light traps in sandy areas with *Acacia* woodland at low elevation (451 m) in southwestern Saudi Arabia.

#### Pseudomallada spp.

Taxon classificationAnimaliaNeuropteraChrysopidae

A83AD498-7C08-5569-B97D-8EB8D7690B31

##### Material examined.

**Al Bahah Province**: Al Mekhwah, Shada Al-A’Ala Nature Reserve, 19°50'51"N, 41°18'06"E, 1358 m, 9 Apr 2019, LT, D. Baiocchi et al. leg., 1♀ and 1♂; *ibidem*, 19°50.329'N, 41°18.604'E, 1563 m, 29 Mar 2017, ST, 1♂; Wadi Reyam (NE Al Makhwah), 19°50'28"N, 41°22'34"E, 473 m, 7 Apr 2019, LT, D. Baiocchi et al. leg., 3♀, 1♂ and 1ex. **Asir Province**: Abha, Wadi Marabah (WSW Abha), 1 Apr 2017, LT, M.S. Abdel-Dayem leg., 1♀; 18°10.293'N, 42°22.195'E, 1150 m; *ibidem*, 4 Apr 2017, LT, M.S. Abdel-Dayem leg., 2♀ and 2♂. Khamis Mushait, Wadi Ibn Hashbal (14 km N Khamis Mushait), 18°27'34"N, 42°42'53"E, 1926 m, 2 Apr 2017, LT, D. Baiocchi leg., 2♀. Rijal Almaa, Wadi Kasan (2 km N Al Hubail), 18°07.12'N, 42°13.55'E, 489 m, 5 Apr 2017, LT, D. Baiocchi leg., 4♀; *ibidem*, 1♀ (AL). **Jizan Province**: Al Darb, Wadi Reem, 17°52.551'N, 42°16.664'E, 136 m, 5 Apr 2017, LT, M.S. Abdel-Dayem leg., 21♀ and 12♂, 1♀; *ibidem*, 1♂ (AL).

##### Notes.

*Pseudomallada* Tsukaguchi 1995 is one of the most species-rich genera within the green lacewing family Chrysopidae and is one of the largest in the order Neuroptera (Duelli et al. 2017). Hölzel (1980, 1988, 1995) described several new species of this genus in the Arabian Peninsula; however, a revision of species in this zoogeographic area is not available, with species identification of specimens often being difficult in preserved alcohol (as they are not always in good condition). Specimens were collected at elevations of 136–1926 m in southwestern Saudi Arabia. The listed adult specimens were captured in sugar traps in rocky areas with a Barbary fig shrub community, and by light traps in rocky and sandy areas with *Acacia* woodlands.

#### Chrysoperla carnea

Taxon classificationAnimaliaNeuropteraChrysopidae

s. l. (Stephens, 1836)

30DD0044-98B4-5F39-BCBC-E237F68AFE79

##### Material examined.

**Al Bahah Province**: Al Mandaq, Wadi Tourabah (E An Na’Amah), 20°11'01"N, 41°18'42"E, 1826 m, 6 Apr 2019, LT, D. Baiocchi et al. leg., 1♀ and 1♂. **Asir Province**: Abha, Wadi Marabah (WSW Abha, near Wadi Mashwas), 18°10.293'N, 42°22.195'E, 1150 m, 1 Apr 2017, LT, M.S. Abdel-Dayem leg., 2♀; *ibidem*, 18°10.18'N, 42°22.12'E, 1197 m, 1 Apr 2017, LT, D. Baiocchi leg., 1♀. **Jizan Province**: Al Darb, Wadi Reem, 17°52.551'N, 42°16.664'E, 136 m, 5 Apr 2017, LT, M.S. Abdel-Dayem leg., 3♀ and 2♂. **Riyadh Province**: Hawtat Bani Tamim, Ibex Reserve Protected Area (W Hotat Bani Tamim), 23°2107'N, 46°21.36'E, 709 m, 11 Apr 2017, LT, D. Baiocchi leg., 1♂.

##### Distribution.

This species is widely distributed in the Palaearctic region, extending to Afrotropical (Cape Verde, Oman, United Arab Emirates, Yemen) and Oriental (China, India, Nepal) regions.

##### Notes.

As reported by Hölzel (2002), knowledge about species, as well as the subspecies of *C. phaenon* in the *carnea*-group, of populations in the Arabian Peninsula remains unresolved. Hölzel (1980) recorded this species in the Eastern Province, Madinah, Makkah, and Riyadh provinces of Saudi Arabia. The listed specimens were collected from southwestern and central parts of Saudi Arabia, at elevations up to 1197 m. Most specimens were collected from rocky and sandy areas with *Acacia* woodlands.

#### Brinckochrysa alfierii

Taxon classificationAnimaliaNeuropteraChrysopidae

(Navás, 1926)

956D6410-2911-5CE5-91AD-BEE544A5FE42

##### Material examined.

**Jizan Province**: Al Darb, Wadi Reem, 17°52.551'N, 42°16.664'E, 136 m, 5 Apr 2017, LT, M.S. Abdel-Dayem leg., 1♂.

##### Distribution.

Africa: Algeria, Egypt, Eritrea, Libya, Sudan, Tunisia. Asia: Israel, Oman, Saudi Arabia, Yemen. A polycentric Afro-syroeremic species.

##### Biology.

Practically unknown. Adults were collected on *Tamarix* sp. in sand dune and coastal dune habitats (Hölzel 2002).

##### Notes.

This species was previously reported in Riyadh Province (Hölzel 1980). The listed single male specimen was attracted to a light trap in sandy areas with *Acacia* woodlands at low elevation of 136 m in southwestern Saudi Arabia.

#### Brinckochrysa chlorosoma

Taxon classificationAnimaliaNeuropteraChrysopidae

(Navás, 1914)

E8B67CF8-48D9-5529-AD62-667BBD69DA7B

##### Material examined.

**Al Bahah Province**: Al Mandaq, Wadi Tourabah (E An Na’Amah), 20°11'01"N, 41°18'42"E, 1826 m, 6 Apr 2019, LT, D. Baiocchi et al. leg., 1♂. Al Makhwah, Wadi Reyam (NE Al Makhwah), 19°50'28"N, 41°22'34"E, 473 m, 7 Apr 2019, LT, D. Baiocchi et al. leg., 1♀ and 1♂;

##### Distribution.

Africa: widespread, Cabo Verde. Asia: Israel, Oman, Saudi Arabia, Yemen. Europe: Greece, Italy, Malta. An eremic Afrotropical species.

##### Notes.

This species was previously reported in Makkah Province (Hölzel 1980). The listed specimens were collected in sandy areas with *Acacia* woodlands at elevations of 47‒1826 m in southwestern Saudi Arabia.

#### Chrysemosa andresi

Taxon classificationAnimaliaNeuropteraChrysopidae

(Navás, 1915)

FFE6421D-3094-563B-B6FA-FCAE80C30DC8

##### Material examined.

**Al Bahah Province**: Al Mandaq, Wadi Tourabah (E An Na’Amah), 20°11'01"N, 41°18'42"E, 1826 m, 6 Apr 2019, LT, D. Baiocchi et al. leg., 2♀. Al Mekhwah, Shada Al-A’Ala Nature Reserve, 19°51.682'N, 41°18.263'E, 1291 m, 29 Mar 2017, LT, M.S. Abdel-Dayem leg., 1♀; *ibidem*, 19°50'51"N, 41°18'06"E, 1358 m, 9 Apr 2019, LT, D. Baiocchi et al. leg., 4♀ and 2♂; *ibidem*, 19°50.329'N, 41°18.604'E, 1563 m, 29 Mar 2017, ST, 1♂; 10 km NNW of Al Makhwah, 20°10.750'N, 41°19.072'E, 554 m, 30 Mar 2017, LT, M.S. Abdel-Dayem leg., 2♂; Wadi Reyam (NE Al Makhwah), 19°50'28"N, 41°22'34"E, 473 m, 7 Apr 2019, LT, D. Baiocchi et al. leg., 7♀ and 8♂. **Asir Province**: Abha, Wadi Marabah (WSW Abha), 18°10.293'N, 42°22.195'E, 1150 m, 1 Apr 2017, LT, M.S. Abdel-Dayem leg., 3♀ and 3♂, 1♀; *ibidem*, 1♂ (AL); *ibidem*, 4 Apr 2017, LT, M.S. Abdel-Dayem leg., 1♀; *ibidem*, 18°10.18'N, 42°22.12'E, 1197 m, 1 Apr 2017, LT, D. Baiocchi leg., 2♀. Khamis Mushait, Wadi Ibn Hashbal (14 km N Khamis Mushait), 18°27.34'N, 42°42.53'E, 1926 m, 2, Apr 2017, LT, D. Baiocchi leg., 1♀ and 1♂. Rijal Almaa, Wadi Kasan (3 km N Al Hubail), 18°06.981'N, 42°13.939'E, 451 m, 3 Apr 2017, LT, M.S. Abdel-Dayem and I. Rasool leg., 1♀. **Jizan Province**: Al Darb, Wadi Reem, 17°52.551'N, 42°16.664'E, 136 m, 5 Apr 2017, LT, M.S. Abdel-Dayem leg., 3♀ and 1♂.

##### Distribution.

Africa: Algeria, Egypt, Senegal, Sudan. Asia: Iran, Oman, Saudi Arabia, Yemen. A polycentric Afro-syro-iranoeremic species.

##### Notes.

*Chrysemosa andresi* was recorded in Asir Province (Hölzel 1988). The specimens were collected at different elevations (136–1926 m) in southwestern Saudi Arabia. The listed specimens were caught by sugar traps set in rocky Barbary fig shrub communities and by light traps set in rocky and sandy areas with *Acacia* woodlands.

### Hemerobiidae Latreille, 1802

#### Micromus sjostedti

Taxon classificationAnimaliaNeuropteraHemerobiidae

van der Weele, 1910

A1C98BB9-8665-51D9-8B6E-B3CD4FE7A6C6

##### Material examined.

**Jizan Province**: Al Darb, Wadi Reem, 17°52.551'N, 42°16.664'E, 136 m, 5 Apr 2017, LT, M.S. Abdel-Dayem leg., 1♀.

##### Distribution.

Africa: sub-Saharan Africa (widespread) to South Africa, Cabo Verde. Asia: Saudi Arabia, Yemen. A possible Afrotropical species.

##### Notes.

The species was previously documented in Asir Province (Hölzel 1988). The listed female specimen was collected at low elevation (136 m) in southwestern Saudi Arabia in a sandy area with *Acacia* woodlands.

### Mantispidae Leach in Brewster, 1815

#### Afromantispa nana

Taxon classificationAnimaliaNeuropteraMantispidae

(Erichson, 1839)

4064D68A-43F3-5C27-B3BE-AB65482FAE35

##### Material examined.

**Al Bahah Province**: Al Mandaq, Wadi Tourabah (E An Na’Amah), 20°11'01"N, 41°18'42"E, 1826 m, 6 Apr 2019, LT, D. Baiocchi et al. leg., 6♀ and 5♂. Al Mekhwah, Shada Al-A’Ala Nature Reserve, 19°50.51'N, 41°18.06'E, 1358 m, 9 Apr 2019, LT, D. Baiocchi et al. leg., 10♀, 11♂ and 1 ex; *ibidem*, 14 Apr 2016, LT, D. Baiocchi leg., 1♀ and 1♂ (AL); Wadi Reyam (NE Al Makhwah), 19°50'28"N, 41°22'34"E, 473 m, 7 Apr 2019, LT, D. Baiocchi et al. leg., 6♀, and 24♂. **Asir Province**: Abha, Wadi Marabah (WSW Abha), 18°10.18'N, 42°22.12'E, 1197 m, 11‒13 Apr 2019, LT, D. Baiocchi et al. leg., 2♀ and 1♂; *ibidem*, 16 Apr 2016, LT, D. Baiocchi leg., 1♀ and 1♂ (AL). Khamis Mushait, Wadi Ibn Hashbal (14 km N Khamis Mushait), 18°27.34'N, 42°42.53'E, 1926 m, LT, 2 Apr 2017, LT, D. Baiocchi leg., 1♀; *ibidem*, 2 Apr 2017, LT, M.S. Abdel-Dayem leg., 1♂. Rijal Almaa, Wadi Kasan (3 km N Al Hubail), 18°06.981'N, 42°13.939'E, 451 m, 3 Apr 2017, LT, M.S. Abdel-Dayem and I. Rasool leg., 1♀; *ibidem*, 18°07.12'N, 42°13.55'E, 489 m, 5 Apr 2017, LT, D. Baiocchi leg., 1♀; *ibidem*, 18°06'57"N, 42°13'55"E, 462 m, 12 Apr 2019, LT, D. Baiocchi et al. leg., 1♂. **Jizan Province**: Al Darb, Wadi Reem, 17°52.551'N, 42°16.664'E, 136 m, 5 Apr 2017, LT, M.S. Abdel-Dayem leg., 2♀ and 2♂.

##### Distribution.

Africa: Burkina Faso, Djibouti, Eritrea, Republic of the Congo, South Africa, Sudan. Asia: Saudi Arabia, United Arab Emirates, Yemen. A possible Afrotropical species.

##### Biology.

**Unknown.**

##### Notes.

This species was previously recorded in the Arabian Peninsula (Yemen: Aden) as *Necyla arabica* (Navás 1914), now a junior synonym. The listed specimens were collected in rocky and sandy areas with *Acacia* woodlands at different elevations (136–1926) in southwestern Saudi Arabia.

#### Mantispa aphavexelte

Taxon classificationAnimaliaNeuropteraMantispidae

Aspöck & Aspöck, 1994

AECF3F43-B18E-5B3F-8EE5-41C0484EAEB2

##### Material examined.

**Asir Province**: Abha, Wadi Marabah (WSW Abha), 18°10.18'N, 42°22.12'E, 1197 m, 1 Apr 2017, LT, D. Baiocchi leg., 1♂; *ibidem*, 1♂ (AL); *ibidem*, 11‒13 Apr 2019, LT, D. Baiocchi et al. leg., 1♂.

##### Distribution.

Africa: Morocco. Asia: Armenia, China[?], Iran, Kazakhstan, Mongolia, Russia, Turkey. Europe: widespread in southern Europe. It is a Palearctic species.

##### Biology.

*Mantispa aphavexelte* was previously found in ruderal areas and olive groves. The larvae parasitize spiders and feed on spider eggs (Aspöck et al. 1980).

##### Notes.

This study presents the first report for this species in Saudi Arabia. The three males were collected from mountainous *Acacia* woodlands at an elevation of 1197 m in southwestern Saudi Arabia.

### Berothidae Handlirsch, 1908

#### Nodalla eatoni

Taxon classificationAnimaliaNeuropteraBerothidae

(McLachlan, 1898)

A35E7B0B-A013-518E-A29B-392B70C6F00C

##### Material examined.

**Al Bahah Province**: Al Mekhwah, Shada Al-A’Ala Nature Reserve, 19°51.066'N, 41°18.037'E, 1325 m, 2 Nov 2015, LT, Al Dhafer et al. leg., 2♀; 10 km NNW of Al Makhwah, 20°10.750'N, 41°19.072'E, 554 m, 30 Mar 2017, LT, M.S. Abdel-Dayem leg., 1♀.

##### Distribution.

Africa: widely distributed in northern Africa. Asia: Israel, Oman, Saudi Arabia, Yemen. A polycentric Afro-syroeremic species.

##### Biology.

*Nodalla eatoni* is found in semi-deserts with sparse low vegetation habitats, mainly in the form of isolated spiny dwarf shrubs, surrounded by extensive vegetation-free sandy areas (Aspöck and Aspöck 1983).

##### Notes.

The species was previously documented in several localities (Aspöck and Aspöck 1998). The listed female specimen was collected in foothill *Acacia* woodlands at elevation of 554‒1325 m in southwestern Saudi Arabia.

#### Nodalla saharica

Taxon classificationAnimaliaNeuropteraBerothidae

(Esben-Petersen, 1920)

EC43D44D-F0AA-5965-B63E-89A7A39BC28E

##### Material examined.

**Al Bahah Province**: Al Mekhwah, Shada Al-A’Ala Nature Reserve, 19°51.006'N, 41°18.037'E, 1325 m, 5 Mar 2015, LT, Al Dhafer et al. leg., 2♂; *ibidem*, 2 Nov 2015, LT, Al Dhafer et al. leg., 1♀ and 1♂; *ibidem*, 19°50'51"N, 41°18'06"E, 1358 m, 9 Apr 2019, LT, D. Baiocchi et al. leg., 1♀ and 1♂; *ibidem*, 19°50.329'N, 41°18.604'E, 1563 m, 2 Nov 2015, LT, Al Dhafer et al. leg., 2♀. **Asir Province**: Abha, Garf Raydah Nature Reserve, 18°11.749'N, 42°23.345'E, 1614 m, 7 May 2015, LT, Al Dhafer et al. leg., 2♀ and 1♂; *ibidem*, 18°11.695'N, 42°23.818'E, 1897 m, 5 Nov 2015, LT, Al Dhafer et al. leg., 1♀; Wadi Marabah (WSW Abha), 18°10.293'N, 42°22.195'E, 1150 m, 1 Apr 2017, LT, M.S. Abdel-Dayem leg., 1♀; *ibidem*, 4 Apr 2017, LT, M.S. Abdel-Dayem leg., 1♂.

##### Distribution.

Africa: throughout northern Africa. Asia: Afghanistan, Iran, Iraq, Israel, Oman, Saudi Arabia, Yemen. A polycentric Afro-syro-iranoeremic species.

##### Biology.

The biology of *N. saharica* is largely unknown, as with other *Nodalla* species. Adults hide deep inside sparse vegetation or crevices and under stones during the day; at night they are attracted to artificial light sources (Aspöck and Aspöck 1998).

##### Notes.

The species was formerly reported in several localities (Aspöck and Aspöck 1998). The listed specimens were collected in mountainous *Acacia* woodlands, Barbary fig shrublands, and *O. europaea* communities at different elevations (1150‒1897 m) in southwestern Saudi Arabia.

#### Podallea arabica

Taxon classificationAnimaliaNeuropteraBerothidae

Aspöck & Aspöck, 1981

F8490AA6-50E1-508D-8A6E-B5A57354F8EC

##### Material examined.

**Al Bahah Province**: Al Mekhwah, Shada Al-A’Ala Nature Reserve, 19°50'51"N, 41°18'06"E, 1358 m, 9 Apr 2019, LT, D. Baiocchi et al. leg., 1♂. **Asir Province**: Abha, Wadi Marabah (WSW Abha), 18°10.18'N, 42°22.12'E, 1197 m, 16 Apr 2016, LT, D. Baiocchi leg., 1♂ (AL). **Jizan Province**: Al Darb, Wadi Reem, 17°52.551'N, 42°16.664'E, 136 m, 5 Apr 2017, LT, M.S. Abdel-Dayem leg., 1♀.

##### Distribution.

Endemic to Saudi Arabia.

##### Notes.

The species was previously recorded in Asir Province (Aspöck and Aspöck 1981). The listed adult specimens were collected in sandy and rocky areas with *Acacia* woodlands at elevations of 136‒1359 m in southwestern Saudi Arabia.

### Nemopteridae Burmeister, 1839

#### Croce aristata

Taxon classificationAnimaliaNeuropteraNemopteridae

(Klug, 1836)

5A733A99-DEC8-5118-BAD7-713522521B10

##### Material examined.

**Al Bahah Province**: Al Makhwah, Wadi Reyam (NE Al Makhwah), 19°50'28"N, 41°22'34"E, 473 m, 7 Apr 2019, LT, D. Baiocchi et al. leg., 1♀.

##### Distribution.

Africa: widespread in northern Africa, Ethiopia. Asia: Israel, Oman, Saudi Arabia. Polycentric Afro-syroeremic species.

##### Biology.

This species lives in deserted mines and caves, normally hiding under stones (Hafez and El Moursy 1964).

##### Notes.

It was previously collected in Riyadh Province (Meinander 1980). The listed female specimen was collected in foothill *Acacia* woodlands at low elevation of 473 m in southwestern Saudi Arabia.

#### Dielocroce berlandi

Taxon classificationAnimaliaNeuropteraNemopteridae

(Navás, 1936)

80558151-2225-56E9-B6C0-42798BA053FB

##### Material examined.

**Al Bahah Province**: Al Makhwah, Wadi Reyam (NE Al Makhwah), 19°50'28"N, 41°22'34"E, 473 m, 7 Apr 2019, LT, D. Baiocchi et al. leg., 1♀. **Asir Province**: Rijal Almaa, Wadi Kasan (3 km N Al Hubail), 18°06'57"N, 42°13'55"E, 462 m, 12 Apr 2019, LT, D. Baiocchi et al. leg., 5♀.

##### Distribution.

Africa: spread throughout North Africa, Kenya, Sudan. Asia: Israel, Saudi Arabia, Yemen. Polycentric Afro-syroeremic species.

##### Notes.

It was previously collected in Al Madinah Province (Meinander 1980). The listed specimens were found in *Acacia* woodlands at low elevations of 462‒473 m in southwestern Saudi Arabia.

#### Dielocroce chobauti

Taxon classificationAnimaliaNeuropteraNemopteridae

(McLachlan, 1898)

25527866-F62D-5A0F-8C9F-A43B11F0CE9E

##### Material examined.

**Al Bahah Province**: Al Mekhwah, Shada Al Asfal, Al-Hamadah, 20°10.750'N, 41°19.072'E, 554 m, 30 Mar 2017, LT, M.S. Abdel-Dayem leg., 1♀.

##### Distribution.

Africa: widespread in North Africa, Sudan, Somalia. Asia: Israel, Oman, Saudi Arabia, Yemen. A possible polycentric Afro-syroeremic species.

##### Notes.

It was previously collected in Asir, Hail and Makkah provinces (Meinander 1980). The listed female specimen was collected in foothill *Acacia* woodlands at an elevation of 554 m in southwestern Saudi Arabia.

#### Dielocroce elegans

Taxon classificationAnimaliaNeuropteraNemopteridae

(Alexandrov-Martynov, 1930)

A80B07C2-EC81-5F27-8E3D-BC0EA3769552

##### Material examined.

**Al Bahah Province**: Al Mekhwah, Shada Al Asfal, Al-Hamadah, 20°10.750'N, 41°19.072'E, 554 m, 30 Mar 2017, LT, M.S. Abdel-Dayem leg., 1♂; 10 km NNW of Al Makhwah, 19°50.47'N, 41°22.40'E, 630 m, 31 Mar 2017, LT, D. Baiocchi leg., 5♀ and 4♂. **Riyadh Province**: Hotat Bani Tamim, Ibex Reserve Protected Area, (W Hotat Bani Tamim), 23°21.07'N, 46°21.36'E, 709 m, 11 Apr 2017, LT, D. Baiocchi leg., 33♀ and 26♂; *ibidem*, 1♀ and 1♂ (AL). Riyadh, NW Al Uyaynah, 24°53.33'N, 46°17.40'E, 761 m, 10 Apr 2016, LT, D. Baiocchi leg., 2♀; *ibidem*, 1♀ (AL).

##### Distribution.

Asia: Afghanistan, Iran, Israel, Oman, Pakistan, Saudi Arabia, Syria, United Arab Emirates, Yemen. A Syro-iranoeremic species.

##### Notes.

This species was formerly reported in Asir, Makkah and Riyadh provinces (Meinander 1980). The listed specimens were collected in sandy areas with *Acacia* woodlands at low elevation (554‒6761 m) in southwestern and central Saudi Arabia.

#### Dielocroce

Taxon classificationAnimaliaNeuropteraNemopteridae

sp.

6543CE3A-9C1D-570A-899D-A91FED34CBC5

##### Material examined.

♀. **Riyadh Province**: Hotat Bani Tamim, Ibex Reserve Protected Area, (W Hotat Bani Tamim), 23°21.07'N, 46°21.36'E, 709 m, 11 Apr 2017, LT, D. Baiocchi leg., 1♀.

##### Notes.

This listed female specimen was collected at an elevation of 709 m among a huge number of *D. elegans* in sandy *Acacia* woodlands at low elevation in central Saudi Arabia. It might be *D. berlandi* (Navás 1936), but the poor condition of the specimen preserved in alcohol resulted in our identification only to the genus level.

#### Halter halteratus

Taxon classificationAnimaliaNeuropteraNemopteridae

(Forskål, 1775)

5F03C30C-B524-558F-A23A-006C03E62F27

##### Material examined.

**Riyadh Province**: Al Aflag, Farshet Sheaal (NW Al Naifiyah), 22°25.496'N, 46°34.544'E, 606 m, LT, 10 Apr 2015, LT, Al Dhafer et al. leg., 1♀; *ibidem*, 22°24.381'N, 46°35.594'E, 596 m, LT, 12 Apr 2015, LT, Al Dhafer et al. leg., 1♂; Wadi Ghaihab (33 km N Layla), 22°19.601'N, 46°24.808’E, 460 m, LT, 10 Apr 2015, LT, Al Dhafer et al. leg., 1♂. Al Zulfi, Rawdhat Al Sabalah, 26°21.522'N, 44°59.011'E, 664 m, LT, 19 May 2015, LT, Al Dhafer et al. leg., 2♂. Hotat Bani Tamim, Ibex Reserve Protected Area, (W Hotat Bani Tamim), 23°21.07'N, 46°21.36'E, 709 m, 11 Apr 2017, LT, D. Baiocchi leg., 2♀ and 9♂; *ibidem*, 1♀ and 1♂ (AL). Ramah, Rawdat Khuraim (100 km NE Riyadh), 25°25.943'N, 47°13.863'E, 572 m, 15 May 2012, LT, M.S. Abdel-Dayem leg., 1♀ and 1♂; *ibidem*, 25°22.986'N, 47°16.712'E, 559 m, 28 Apr 2012, LT, M.S. Abdel-Dayem leg., 3♂. Riyadh, Wadi Hanifa, 24°54.422'N, 46°10.903'E, 809 m, LT, 22 Apr 2017, M. Abdel-Dayem et al. leg., 3♂.

##### Distribution.

Africa: widespread in North Africa, Mauritania, Sudan. Asia: Afghanistan, India, Iran, Iraq, Israel, Kuwait, Oman, Pakistan, Saudi Arabia, Syria, Yemen. A polycentric Afro-syro-iranoeremic species.

##### Notes.

This species was previously collected in Hail, Madinah, Makkah, and Riyadh provinces (Meinander 1980). The listed specimens were collected at low elevations (460‒809 m) in central Saudi Arabia in sandy areas dominated with *Acacia* woodlands or *Calotropis procera* (Aiton) W.T. Aiton (Apocynaceae), or areas cultivated with wheat. Also, two males were collected from *Acacia gerrardii* Benth. (Fabaceae), in a gravelly area at Wadi Ghaihab, Al Aflag.

### Myrmeleontidae Latreille, 1802

#### Goniocercus walkeri

Taxon classificationAnimaliaNeuropteraMyrmeleontidae

(McLachlan, 1894) (Fig. 3A)

3AD70EB7-5521-5C5C-8356-132288887433

##### Material examined.

**Al Bahah Province**: Al Mekhwah, Shada Al-A’Ala Nature Reserve, 19°52.596'N, 41°18.672'E, 892 m, 21 Apr 2014, LT, Al Dhafer et al., 1♀; Wadi Reyam (NE Al Makhwah), 19°50'28"N, 41°22'34"E, 473 m, 7 Apr 2019, LT, D. Baiocchi et al. leg., 1♀ and 1♂. **Riyadh Province**: Al Aflag, Farshet Sheaal (NW Al Naifiyah), 22°25.543'N, 46°34.543'E, 589 m, 15 Oct 2015, LT, Al Dhafer et al. leg., 1♀. Ramah, Rawdat Khuraim (100 km NE Riyadh), 25°25.943'N, 47°13.863'E, 572 m, 28 Aug 2012, LT, M.S. Abdel-Dayem et al. leg., 1♂.

##### Distribution.

Arica: Kenya, Sudan. Asia: Iran, Israel, Saudi Arabia, Yemen. A polycentric Afro-syro-iranoeremic species.

##### Notes.

This species was previously reported in Al Bahah Province (Hölzel 1982). The listed specimens were collected in sandy *Acacia* woodlands at elevations of 473‒892 m in southwestern Saudi Arabia, and from communities of *Acacia ehrenbergiana* Heyne and *Rhazya stricta* Decne. (Apocynaceae) at elevations of 572‒589 m in the sandy areas of central Saudi Arabia.

#### Stenares irroratus

Taxon classificationAnimaliaNeuropteraMyrmeleontidae

Navás, 1912 (Fig. 3B)

02A8EA42-B80C-593B-9691-E916A363252A

##### Material examined.

**Al Bahah Province**: Al Mekhwah, Shada Al-A’Ala Nature Reserve, 19°52.596'N, 41°18.672'E, 892 m, 23 Aug 2014, LT, Al Dhafer et al. leg. 2♂. **Asir Province**: Rijal Almaa, Wadi Kasan (3 km N Al Hubail), 18°07.12'N, 42°13.55'E, 467 m, 5 Apr 2017, LT, 1♀ and 1♂. **Jizan Province**: Al Darb, Wadi Reem, 17°52.551'N, 42°16.664'E, 136 m, 5 Apr 2017, LT, M.S. Abdel-Dayem leg., 1♀.

##### Distribution.

Africa: Egypt. Asia: Israel, Oman, Saudi Arabia, Yemen. A Syroeremic species.

##### Notes.

This species was previously reported in Makkah Province (Hölzel 1988). The listed specimens were collected in sandy areas with *Acacia* woodlands at low elevations (16‒892 m) in southwestern Saudi Arabia.

#### Fadrina formosa

Taxon classificationAnimaliaNeuropteraMyrmeleontidae

(Hölzel, 1981)

6630C462-6573-5C45-86E4-3C3D1A2E5A1E

##### Material examined.

**Al Bahah Province**: Al Mekhwah, Wadi Reyam (NE Al Makhwah), 19°50'28"N, 41°22'34"E, 473 m, 7 Apr 2019, LT, D. Baiocchi et al. leg., 1♀.

##### Distribution.

Africa: Egypt, Sudan. Asia: Israel, Oman, Saudi Arabia, Yemen. An Afro-syroeremic species.

##### Notes.

This species was previously reported in Asir Province (Hölzel 1982). The listed female specimen was collected in sandy areas with *Acacia* woodlands at low elevation of 473 m in southwestern Saudi Arabia.

#### Centroclisis speciosa

Taxon classificationAnimaliaNeuropteraMyrmeleontidae

Hölzel, 1983 (Fig. 3C)

76024732-7B32-5DB3-B374-A5344C42AA47

##### Material examined.

**Asir Province**: Abha, Garf Raydah Nature Reserve, 18°11.695'N, 42°23.818'E, 1897 m, 28 Apr 2014, LT, Al Dhafer et al. leg., 1♀.

##### Distribution.

Asia: Oman, Saudi Arabia, United Arab Emirates, Yemen. Endemic to the Arabian Peninsula.

##### Notes.

*C. speciosa* has been widely reported in Saudi Arabia (Saji and Whittington 2008) and was documented in Asir Province (Hölzel 1983). The listed female specimen was collected at an elevation of 1897 m in an *Olea europaea* (Wall. ex G. Don) Cifferi community.

#### Myrmecaelurus lepidus

Taxon classificationAnimaliaNeuropteraMyrmeleontidae

(Klug in Ehrenberg, 1834)

CF392B19-0FF6-5724-BBFD-22F62CA4ABBC

##### Material examined.

**Riyadh Province**: Hotat Bani Tamim, Ibex Reserve Protected Area, (W of Hotat Bani Tamim), 23°21.07'N, 46°21.36'E, 709 m, 11 Apr 2017, LT, D. Baiocchi leg., 2♀ and 2♂; *ibidem*, 1♀ and 2♂ (AL).

##### Distribution.

Africa: Algeria, Egypt, Libya, Tunisia. Asia: Oman, Saudi Arabia. A polycentric Afro-syroeremic species.

##### Biology.

This species is poorly known and is usually reported in savannah habitats (Güsten 2002).

##### Notes.

Hölzel (1982) reported this species for the Riyadh Province. The listed adult specimens were collected at an elevation of 709 m in sandy areas with *Acacia* woodlands in central Saudi Arabia.

#### Iranoleon arabicus

Taxon classificationAnimaliaNeuropteraMyrmeleontidae

Hölzel, 1982

0877FF4D-3F4C-59CE-8CA0-12E62903E282

##### Material examined.

**Riyadh Province**: Hotat Bani Tamim, Ibex Reserve Protected Area, (W of Hotat Bani Tamim), 23°21.07'N, 46°21.36'E, 709 m, 11 Apr 2017, LT, D. Baiocchi leg., 8♀ and 14♂; *ibidem*, 2♀ and 2♂ (AL).

##### Distribution.

Saudi Arabia, United Arab Emirates. An endemic species to the Arabian Peninsula.

##### Notes.

This record is one of the five published localities where this species was collected in Riyadh Province (Hölzel 1982), with large numbers of specimens being preserved in collections. The listed adult specimens were collected at an elevation of 709 m in sandy areas with *Acacia* woodlands in central Saudi Arabia.

#### Lopezus fedtschenkoi

Taxon classificationAnimaliaNeuropteraMyrmeleontidae

(McLachlan in Fedchenko, 1875)

3AF6FAD0-99B6-5BAC-A3B4-CFE59F53A438

##### Material examined.

**Riyadh Province**: Al Quwaiiyah, Rawdhat Al Harmaliyah, 24°17.433'N, 45°08.493'E, 796 m, 17 Apr 2015, LT, M.S. Abdel-Dayem et al. leg. 1♂; 24°17.864’N, 45°08.746’E, 786 m, 19 Apr 2015, PT, M.S. Abdel-Dayem et al. leg. 1♂.

##### Distribution.

Widespread in southern Palearctic region. Africa: Algeria, Tunisia. Asia: Saudi Arabia. An Afro-syroeremic species.

##### Biology.

*Lopezus fedtschenkoi* is associated with desert biotopes.

##### Notes.

It was reported in Riyadh Province (Hölzel 1982). These specimens were collected from sandy areas with *Acacia* (*Acacia gerrardii* and *A. ehrenbergiana*) woodlands at elevations of 786‒796 m in central Saudi Arabia.

#### Gepus invisus

Taxon classificationAnimaliaNeuropteraMyrmeleontidae

Navás, 1912 (Fig. 3E)

0A042A78-5CFA-59E0-99FC-F81C47630C7F

##### Material examined.

**Riyadh Province**: Al Quwaiiyah, Rawdhat Al Harmaliyah, 24°17.433’N, 45°08.493'E, 796 m, 25 Aug 2015, SW, M.S. Abdel-Dayem et al. leg. 1♂. Al Zulfi, Rawdhat Al-Sabalah, 26°22.056'N, 44°59.136'E, 671 m, 29 Aug 2015, PT, M.S. Abdel-Dayem et al. leg. 1♂.

##### Distribution.

Africa: Algeria, Egypt, Libya, Mauritania, Morocco, Sudan, Tunisia. Asia: Israel, Iran, Iraq, Oman, Saudi Arabia, United Arab Emirates, Yemen. A polycentric Afro-syro-iranoeremic species.

##### Biology.

This species is associated with desert biotopes.

##### Notes.

This species was reported in the Eastern, Madinah, and Riyadh provinces (Hölzel 1982). The listed male specimens were collected at elevations of 671‒796 m in sandy areas with milkweed trees, *Calotropis procera*, and sandy areas with *Acacia* woodlands, *Acacia ehrenbergiana*, and *A. gerrardii* Benth. (Fabaceae), in central Saudi Arabia.

#### Solter buettikeri

Taxon classificationAnimaliaNeuropteraMyrmeleontidae

Hölzel, 1982

E36EEAA6-EE76-5A38-8C7B-BB790F28740D

##### Material examined.

**Al Bahah Province**: Al Mekhwah, Shada Al-A’Ala Nature Reserve, 19°50.329'N, 41°18.604'E, 1563 m, 29 Mar 2017, ST, 1♂; Wadi Reyam (NE Al Makhwah), 19°50'28"N, 41°22'34"E, 473 m, 7 Apr 2019, LT, D. Baiocchi et al. leg., 9♀ and 7♂; *ibidem*, 1♀ and 1♂ (AL).

##### Distribution.

An endemic species to Saudi Arabia.

##### Notes.

This study presents the first report of this species after the original description from Riyadh Province, based on two males (Hölzel 1982). The listed specimens were collected by light traps in a sandy area with *Acacia* woodlands at low elevation (473 m) and by sugar traps in a rocky area with a Barbary fig shrub community at high elevation (1563 m) in southwestern Saudi Arabia.

#### Solter propheticus

Taxon classificationAnimaliaNeuropteraMyrmeleontidae

Hölzel, 1981

A3DF183B-F204-5FFD-BDBD-E3A5B49AE0E5

##### Material examined.

**Al Bahah Province**: Al Mekhwah, Shada Al-A’Ala Nature Reserve, 19°50'51"N, 41°18'06"E, 1358 m, 9 Apr 2019, LT, D. Baiocchi et al. leg., 1♂.

##### Distribution.

Africa: Egypt, Sudan. Asia: Israel, Saudi Arabia. An Afro-syroeremic species.

##### Notes.

The species was previously reported in several localities (Hölzel 1998). The listed male specimen was collected with light trap at an elevation of 1358 m in a rocky area with *Acacia* woodlands in southwestern Saudi Arabia.

#### Cueta amseli

Taxon classificationAnimaliaNeuropteraMyrmeleontidae

Hölzel, 1982

151B22AC-39BE-544C-800D-78D6E06EA850

##### Material examined.

**Al Bahah Province**: Al Mekhwah, Shada Al-A’Ala Nature Reserve, 19°52.598'N, 41°18.672'E, 892 m, 23 Aug 2014, LT, Al Dhafer et al. leg., 1♂; *ibidem*, 14 Nov 2015, LT, Al Dhafer et al. leg., 2♀; 19°51.762'N, 41°18.089'E, 1225 m, 23 Oct 2014, LT, Al Dhafer et al. leg., 3♀ and 2♂; *ibidem*, 2 Sep 2015, LT, Al Dhafer et al. leg., 1♀ and 2♂; *ibidem*, 17 Oct 2014, LT, Al Dhafer et al. leg., 4♀ and 3♂; *ibidem*, 15 Nov 2015, LT, Al Dhafer et al. leg., 3♂; *ibidem*, 19°51.066'N, 41°18.037'E, 1325, 23 Aug 2014, LT, Al Dhafer et al. leg., 5♀ and 11♂; *ibidem*, 2 Sep 2015, LT, Al Dhafer et al. leg., 3♀ and 4♂; *ibidem*, 17 Oct 2014, LT, Al Dhafer et al. leg., 1♀ and 1♂; *ibidem*, 19°50'51"N, 41°18'06"E, 1358 m, 9 Apr 2019, LT, D. Baiocchi et al. leg., 1♂; *ibidem*, 19°50.710'N, 41°18.267'E, 1474 m, 23 Aug 2014, LT, Al Dhafer et al. leg., 9♀ and 7♂; *ibidem*, 2 Sep 2015, LT, Al Dhafer et al. leg., 3♀ and 2♂; *ibidem*, 17 Oct 2014, LT, Al Dhafer et al. leg., 1♀ and 2♂; *ibidem*, 19°50.411'N, 41°18.686'E, 1611 m, 23 Aug 2014, LT, Al Dhafer et al. leg., 5♀ and 9♂; *ibidem*, 2 Sep 2015, LT, Al Dhafer et al. leg., 1♀ and 1♂. **Asir Province**: Abha, Garf Raydah Nature Reserve, 18°11.749'N, 42°23.345'E, 1614 m, 24 Feb 2014, LT, Al Dhafer et al. leg., 1♀ and 2♂; *ibidem*, 5 Sep 2015, LT, Al Dhafer et al. leg., 2♀ and 2♂; *ibidem*, 18°11.766'N, 42°24.315'E, 2285 m, 6 Jun 2014, LT, Al Dhafer et al. leg., 1♀; *ibidem*, 31 Jul 2015, LT, Al Dhafer et al. leg., 1♀; Wadi Marabah (WSW Abha), 18°10.18'N, 42°22.12'E, 1197 m, 1 Apr 2017, LT, D. Baiocchi leg., 5♀ and 1♂; *ibidem*, 11–13 Apr 2019, LT, D. Baiocchi et al. leg., 1♂.

##### Distribution.

Oman, Saudi Arabia, Yemen. An endemic species to the Arabian Peninsula.

##### Biology.

Unknown, possibly a pit builder in arid biotopes, like congeners.

##### Notes.

This record is the second locality published for Saudi Arabia, with it being previously collected in Asir Province (Hölzel 1982), with a large number of specimens being preserved in collections. The specimens were collected from mountainous *Acacia* woodlands, Barbary fig shrubland, and *O. europaea* communities at different elevations (892‒2285 m) in southwestern Saudi Arabia.

#### Cueta asirica

Taxon classificationAnimaliaNeuropteraMyrmeleontidae

Hölzel, 1982

E08943E0-2645-5820-9858-2672B36BDED1

##### Material examined.

**Al Bahah Province**: Al Mekhwah, Shada Al-A’Ala Nature Reserve, 19°52.598'N, 41°18.672'E, 892 m, 26 Jan 2015, LT, Al Dhafer et al. leg., 1♂; *ibidem*, 19°50.575'N, 41°18.691'E, 1666 m, 27 Jul 2015, LT, Al Dhafer et al. leg., 1♀. **Asir Province**: Abha, Garf Raydah Nature Reserve, 18°11.749'N, 42°23.345'E, 1614 m, 6 Jun 2014, LT, Al Dhafer et al. leg., 1♀ and 2♂; *ibidem*, 18°11.884'N, 42°24.435'E, 2387 m, 6 Jun 2014, LT, Al Dhafer et al. leg., 1♀; Wadi Marabah (WSW Abha), 18°10.293'N, 42°22.195'E, 1150 m, 1 Apr 2017, M.S. Abdel-Dayem leg., LT, 2♀ and 4♂; *ibidem*, 1♀ and 1♂ (AL); *ibidem*, 18°10.18'N, 42°22.12'E, 1197 m, 1 Apr 2017, LT, D. Baiocchi leg., 7♀ and 8♂. Rijal Almaa, Wadi Kasan (2 km N Al Hubail), 18°07.12'N, 42°13.55'E, 489 m, 5 Apr 2017, LT, D. Baiocchi leg., 1♀.

##### Distribution.

This species is endemic to Saudi Arabia.

##### Biology.

Unknown, possibly a pit builder in arid biotopes, like congeners.

##### Notes.

These records represent new localities, with this species previously being collected in Al Bahah Province (Hölzel 1982), with a large number of specimens being preserved in collections. The listed specimens were collected by light traps in *Acacia* woodlands growing on both sandy and rocky soils, and from Barbary fig shrublands and *O. europaea* communities, at different elevations (489‒2387 m) in southwestern Saudi Arabia.

#### Cueta lineosa

Taxon classificationAnimaliaNeuropteraMyrmeleontidae

(Rambur, 1842)

8287BA29-2121-5C4F-9025-804807D12601

##### Material examined.

**Al Bahah Province**: Al Mekhwah, 10 km NNW of Al Makhwah, 20°10.750'N, 41°19.072'E, 554 m, 30 Mar 2017, LT, S.A. El-Sonbati leg., 2♀ and 1♂; Wadi Reyam (NE Al Makhwah), 19°50'28"N, 41°22'34"E, 473 m, 7 Apr 2019, LT, D. Baiocchi et al. leg., 1♂. **Jizan Province**: Al Darb, Wadi Reem, 17°51.56'N, 42°16.21'E, 139 m, 3 Apr 2017, LT, D. Baiocchi leg., 2♀ and 3♂. **Riyadh Province**: Hotat Bani Tamim, Ibex Reserve Protected Area (W Hotat Bani Tamim), 23°21.07'N, 46°21.36'E, 709 m, 11 Apr 2017, LT, D. Baiocchi leg., 2♀ and 5♂. Ramah, Rawdat Khuraim (100 km NE Riyadh), 25°23.13'N, 47°16.45'E, 550 m, 9 Apr 2016, LT, D. Baiocchi leg., 1♂ (AL).

##### Distribution.

Africa: Egypt, Djibouti, Morocco, Sudan, Tunisia; Asia: Afghanistan, Cyprus, Israel, Iraq, Iran, Lebanon, Oman, Pakistan, Saudi Arabia, Turkey, Turkmenistan, Uzbekistan, Yemen. Europe: Albania, Bulgaria, Greece, Italy, North Macedonia. It is a Palaearctic species.

##### Biology.

This antlion species is common in deserts and steppe-like habitats on the southern edge of the Western Palaearctic. *C. lineosa* larvae construct pits by digging traps in exposed conditions (Badano et al. 2018).

##### Notes.

This species was previously documented in various Saudi provinces, including Asir, Al Bahah, Eastern Province, Jizan, Madinah, and Riyadh (Hölzel 1982). The listed adult specimens were collected in sandy areas with *Acacia* woodlands at low elevations (139‒554 m) in southwestern Saudi Arabia and *Rhazya stricta* communities at elevations of 550‒709 m in central Saudi Arabia.

#### Cueta pallens

Taxon classificationAnimaliaNeuropteraMyrmeleontidae

(Klug in Ehrenberg, 1834)

54DD746B-F5F1-5850-863F-E1A09E50E894

##### Material examined.

**Al Bahah Province**: Al Mekhwah, Shada Al Asfal, Al Hamadah, 20°10.750'N, 41°19.072'E, 554 m, 30 Mar 2017, LT, M.S. Abdel-Dayem leg., 1♀; 10 km NNW of Al Makhwah, 20°10.750'N, 41°19.072'E, 554 m, 30 Mar 2017, LT, S.A. El-Sonbati leg., 1♂. **Asir Province**: Abha, Wadi Marabah (WSW Abha), 18°10.293'N, 42°22.195'E, 1150 m, 1 Apr 2017, LT, M.S. Abdel-Dayem leg., 6♀ and 5♂; *ibidem*, 4 Apr 2017, LT, M.S. Abdel-Dayem leg., 1♀ and 2♂; *ibidem*, 1♀ and 1♂ (AL); *ibidem*, 18°10.18'N, 42°22.12'E, 1197 m, 1 Apr 2017, LT, D. Baiocchi leg., 26♀ and 5♂. Rijal Almaa, Wadi Kasan (2 km N Al Hubail), 18°06.981'N, 42°13.939'E, 451 m, 3 Apr 2017, LT, M.S. Abdel-Dayem and I. Rasool leg., 1♀; *ibidem*, 18°07.12'N, 42°13.55'E, 489 m, 5 Apr 2017, LT, D. Baiocchi leg., 5♀. **Jizan Province**: Al Darb, Wadi Reem, 17°52.551'N, 42°16.664'E, 136 m, 5 Apr 2017, LT, M.S. Abdel-Dayem leg., 1♀. **Riyadh Province**: Hotat Bani Tamim, Ibex Reserve Protected Area, (W Hotat Bani Tamim), 23°21.07'N, 46°21.36'E, 709 m, 11 Apr 2017, LT, D. Baiocchi leg., 2♀ and 1♂.

##### Distribution.

Africa. North Africa (widespread), sub-Saharan Africa, Niger, Madagascar. Asia: Israel, Saudi Arabia, Yemen. A polycentric Afro-syroeremic species.

##### Biology.

*Cueta pallens* is possibly a pit builder in arid biotopes, like congeners.

##### Notes.

This species was previously reported in Makkah and Riyadh provinces (Hölzel 1982). The listed specimens were collected from sandy and rocky areas with *Acacia* woodlands at elevations of 136–1150 m in southwestern and central Saudi Arabia.

#### Myrmeleon caliginosus

Taxon classificationAnimaliaNeuropteraMyrmeleontidae

Hölzel & Ohm, 1983

8BBBBB88-E403-53C5-8EE2-91864B8ED5DC

##### Material examined.

**Asir Province**: Abha, Wadi Marabah (WSW Abha), 18°10.18'N, 42°22.12'E, 1197 m, 1 Apr 2017, LT, D. Baiocchi leg., 1♀.

##### Distribution.

Africa: North Africa (widespread), Cabo Verde (islands: widespread). Asia: Oman, Saudi Arabia, Yemen. An Afrotropical species.

##### Biology.

The larvae are pit-builders that are associated with sandy shorelines and wide, dry sand-covered habitats (Hölzel and Ohm 1983).

##### Notes.

This species was previously collected in Asir and Al Bahah provinces (Hölzel 1988). The listed female specimen was collected from a rocky area with *Acacia* woodlands in the highlands (1197 m elevation) of southwestern Saudi Arabia.

#### Myrmeleon fasciatus

Taxon classificationAnimaliaNeuropteraMyrmeleontidae

(Navás, 1912)

172FDA50-1A9F-5FF6-8CD6-5F404AC36F2B

##### Material examined.

**Asir Province**: Abha, Wadi Marabah (WSW Abha), 18°10.18'N, 42°22.12'E, 1197 m, 11–13 Apr 2019, LT, D. Baiocchi et al. leg., 1♀. Khamis Mushait, Wadi Ibn Hashbal (14 km N Khamis Mushait), 18°27.34'N, 42°42.53'E, 1926 m, 2 Apr 2017, LT, D. Baiocchi leg., 1♂.

##### Distribution.

Africa: northern Africa (widespread). Asia: Israel, Saudi Arabia, Yemen. Europe: Greece. A polycentric Afro-syroeremic species.

##### Biology.

*Myrmeleon fasciatus* inhabits very warm and xeric biotopes, including deserts. The larva builds pits in sheltered areas, such as beneath overhangs and cavities of sedimentary rocks, in very fine detritus or sand (Badano and Pantaleoni 2014).

##### Notes.

This species was previously reported in Al Bahah, Madinah, and Riyadh provinces (Hölzel 1982). The listed specimens were collected from mountainous *Acacia* woodlands at elevations of 1197‒1926 m in southwestern Saudi Arabia.

#### Bankisus maculosus

Taxon classificationAnimaliaNeuropteraMyrmeleontidae

Hölzel, 1983

87D108AB-ACFF-576E-B666-77407D2F8F58

##### Material examined.

**Al Bahah Province**: Al Mekhwah, Shada Al-A’Ala Nature Reserve, 19°51.762'N, 41°18.089'E, 1225 m, 17 Oct 2014, LT, Al Dhafer et al. leg., 2♀; *ibidem*, 19°50.411'N, 41°18.686'E, 1611 m, 20 Apr 2014, LT, Al Dhafer et al. leg., 1♀; **Asir Province**: Abha, Garf Raydah Nature Reserve, 18°11.749'N, 42°23.345'E, 1614 m, 7 May 2015, LT, Al Dhafer et al. leg., 1♂; *ibidem*, 31 Jul 2015, LT, Al Dhafer et al. leg., 1♀; *ibidem*, 18°11.618'N, 42°23.420'E, 1772 m, 31 Jul 2015, LT, Al Dhafer et al. leg., 1♀; *ibidem*, 18°11.679'N, 42°23.691'E, 1851 m, 31 Jul 2015, LT, Al Dhafer et al. leg., 1♂; *ibidem*, 26 Aug 2014, LT, Al Dhafer et al. leg., 1♀, 1♂; 18°11.695'N, 42°23.818'E, 1897 m, 31 Jul 2015, LT, Al Dhafer et al. leg., 2♂; *ibidem*, 18°11.884'N, 42°24.435'E, 2387 m, 31 Jul 2015, LT, Al Dhafer et al. leg., 2♀; Wadi Marabah (WSW Abha, near Wadi Mashwas), 18°10.293'N, 42°22.195'E, 1150 m, 1 Apr 2017, LT, M.S. Abdel-Dayem leg., 1♀; *ibidem*, 4 Apr 2017, LT, M.S. Abdel-Dayem leg., 1♀; *ibidem*, 18°10.18'N, 42°22.12'E, 1197 m, 1 Apr 2017, LT, D. Baiocchi leg., 3♀; 11–13 Apr 2019, LT, D. Baiocchi et al. leg., 1♀ and 1♂; *ibidem*, 16 Apr 2016, LT, D. Baiocchi leg., 2♂ (AL).

##### Distribution.

Asia: Oman, Yemen. An endemic species of the Arabian Peninsula.

##### Notes.

This study presents the first report of the species in Saudi Arabia. The specimens were collected from rocky areas with *Acacia* woodlands, Barbary fig shrublands, and *O. europaea* communities at different elevations (1150‒2387 m) in the mountains of southwestern Saudi Arabia.

#### Omoleon jeanneli

Taxon classificationAnimaliaNeuropteraMyrmeleontidae

Navás, 1936 (Fig. 3D)

4D8321E6-95F7-51B9-9496-7439FD6786F9

##### Material examined.

**Al Bahah Province**: Al Mekhwah, Shada Al-A’Ala Nature Reserve, 19°52.596'N, 41°18.672'E, 892 m, 21 Apr 2014, LT, H. Al Dhafer et al. leg. 1ex.

##### Distribution.

Africa: Ethiopia, Kenya. An Afrotropical species.

##### Notes.

This study presents the first report of the species in Saudi Arabia and Arabian Peninsula. The listed specimen was collected in mountainous *Acacia* woodlands at an elevation of 892 m in southwestern Saudi Arabia.

#### Geyria lepidula

Taxon classificationAnimaliaNeuropteraMyrmeleontidae

(Navás, 1912)

8471659E-6A64-542D-9F68-101F4F1A9136

##### Material examined.

**Riyadh Province**: Ramah, Rawdat Khuraim (100 km NE Riyadh), 25°25.943'N, 47°13.863'E, 572 m, 28 Aug 2012, LT, M.S. Abdel-Dayem leg., 1ex; *ibidem*, 28 Aug 2012, SW (on *Rhazya stricta*), M.S. Abdel-Dayem leg., 1ex; *ibidem*, 24 Sept 2012, LT, M.S. Abdel-Dayem leg., 1ex; *ibidem*, 25°22.986'N, 47°16.712'E, 559 m, 28 Aug 2012, LT, M.S. Abdel-Dayem leg., 1ex; *ibidem*, 9 Sep 2012, LT, M.S. Abdel-Dayem leg., 2ex.

##### Distribution.

Africa: Egypt (including Sinai), Sudan. Asia: India, Iran, Israel, Saudi Arabia, United Arab Emirates. It is an Afro-syro-iranoeremic species.

##### Notes.

This species was previously recorded in Baha, Makkah, and Riyadh provinces (Hölzel, 1982). The listed specimens were collected from sandy areas dominated with *Rhazya stricta* and *Acacia ehrenbergiana* at elevations of 572‒559 m in central Saudi Arabia.

#### Geyria pallida

Taxon classificationAnimaliaNeuropteraMyrmeleontidae

Hölzel, 1983

69DAEDB3-4C49-5FE3-84B0-A54A95E0026D

##### Material examined.

**Riyadh Province**: Hotat Bani Tamim, Ibex Reserve Protected Area, (W Hotat Bani Tamim), 23°21.07'N, 46°21.36'E, 709 m, 11 Apr 2017, LT, D. Baiocchi leg., 1♀; *ibidem*, 1♀ (AL).

##### Distribution.

Saudi Arabia, United Arab Emirates. An Arabian endemic species.

##### Notes.

This species was originally described from Eastern Province (Hölzel 1983). The new locality listed here represents a distributional extension for this species. The specimens were collected in sandy areas with *Acacia* woodlands at an elevation of 709 m in central Saudi Arabia.

#### Neuroleon asirensis

Taxon classificationAnimaliaNeuropteraMyrmeleontidae

Hölzel, 1983

BE72886A-DD16-55C0-BB8A-87E1C9DF235C

##### Material examined.

**Al Bahah Province**: Al Mekhwah, Shada Al-A’Ala Nature Reserve, 19°50'51"N, 41°18'06"E, 1358 m, 9 Apr 2019, LT, D. Baiocchi et al. leg., 2♀ and 2♂. **Asir Province**: Khamis Mushait, Wadi Ibn Hashbal (14 km N Khamis Mushait), 1926 m, 18°27.34'N, 42°42.53'E, 2 Apr 2017, LT, D. Baiocchi leg., 1ex. **Jizan Province**: Al Darb, Wadi Reem, 17°52.551'N, 42°16.664'E, 136 m, 5 Apr 2017, LT, M.S. Abdel-Dayem leg., 2♀.

##### Distribution.

Iran, Oman, Saudi Arabia, United Arab Emirates. A possible Syro-iranoeremic species.

##### Notes.

This species was previously recorded in Asir, Al Bahah, and Makkah provinces (Hölzel 1983). The listed specimens were collected from *Acacia* woodlands in the lowlands and highlands (136‒1926 m elevation) of southwestern Saudi Arabia.

#### Neuroleon delicatus

Taxon classificationAnimaliaNeuropteraMyrmeleontidae

Hölzel, 1983

A21AFF16-8041-544B-BC06-35B530F80E67

##### Material examined.

**Al Bahah Province**: Al Mekhwah, Shada Al-A’Ala Nature Reserve, 19°50.411'N, 41°18.686'E, 1611 m, 17 Nov 2014, LT, Al Dhafer et al. leg., 4♀; Wadi Reyam (NE Al Makhwah), 19°50'28"N, 41°22'34"E, 473 m, 7 Apr 2019, LT, D. Baiocchi et al. leg., 2♀ and 3♂. **Asir Province**: Abha, Garf Raydah Nature Reserve, 18°11.749'N, 42°23.345'E, 1614 m, 20 Oct 2014, LT, Al Dhafer et al. leg., 1♀; *ibidem*, 18°11.679'N, 42°23.691'E, 1851 m, 20 Oct 2014, LT, Al Dhafer et al. leg., 1♀; Wadi Marabah (WSW Abha), 18°10.18'N, 42°22.12'E, 1197 m, 1 Apr 2017, LT, D. Baiocchi leg., 1♀ and 1♂. Rijal Almaa, Wadi Kasan (2 km N Al Hubail), 18°07.12'N, 42°13.55'E, 489 m, 5 Apr 2017, LT, D. Baiocchi leg., 1♀ and 1♂; *ibidem*, 1♀ (AL). **Jizan Province**: Al Darb, Wadi Reem, 17°51.56'N, 42°16.21'E, 139 m, 17°52.551'N, 42°16.664'E, 136 m, 5 Apr 2017, LT, M.S. Abdel-Dayem leg., 1♀; *ibidem*, 3 Apr 2017, LT, D. Baiocchi leg., 1♀.

##### Distribution.

Asia: An endemic species to Saudi Arabia.

##### Notes.

This is the first report of this species since it was first described from Asir and Jizan Provinces (Hölzel 1983). The specimens were collected in sandy and rocky areas with *Acacia* woodlands and Barbary fig shrublands at different elevations (136‒1614 m) in southwestern Saudi Arabia.

#### Neuroleon leptaleus

Taxon classificationAnimaliaNeuropteraMyrmeleontidae

(Navás, 1912)

3F73BEFD-E057-5E2F-B573-FF4D8D0BC12A

##### Material examined.

**Riyadh Province**: Hotat Bani Tamim, Ibex Reserve Protected Area, (W Hotat Bani Tamim), 23°21.07'N, 46°21.36'E, 709 m, 11 Apr 2017, LT, D. Baiocchi leg., 1♂.

##### Distribution.

Africa: Algeria, Libya, Morocco, Tunisia. Asia: Iran, Iraq, Israel, Oman, Saudi Arabia. A polycentric Afro-syro-iranoeremic species.

##### Notes.

It was previously reported in Eastern, Madinah, and Riyadh provinces (Hölzel 1982). The listed specimen was collected from sandy areas with *Acacia* woodlands at an elevation of 709 m in central Saudi Arabia.

#### Neuroleon lugubris

Taxon classificationAnimaliaNeuropteraMyrmeleontidae

(Navás, 1926)

389E0247-0A5E-51C6-94A6-8E837AD35073

##### Material examined.

**Al Bahah Province**: Al Mekhwah, Shada Al-A’Ala Nature Reserve, 19°52.598'N, 41°18.672'E, 892 m, 3 Mar 2015, LT, Al Dhafer et al. leg., 1♂; *ibidem*, 19°51.762'N, 41°18.089'E, 1225 m, 21 Apr 2014, LT, Al Dhafer et al. leg., 2♀; *ibidem*, 19°50'51"N, 41°18'06"E, 1358 m, 9 Apr 2019, LT, D. Baiocchi et al. leg., 2♀; *ibidem*, 19°50.575'N, 41°18.691'E, 1666 m, 20 Apr 2014, LT, Al Dhafer et al. leg., 1♀. **Asir Province**: Abha, Wadi Marabah (WSW Abha), 18°10.293'N, 42°22.195'E, 1150 m, 1 Apr 2017, LT, M.S. Abdel-Dayem leg., 2♀; *ibidem*, 18°10.18'N, 42°22.12'E, 1197 m, 1 Apr 2017, LT, D. Baiocchi leg., 2♀ and 1♂; *ibidem*, 1♀ (AL). Khamis Mushait, Wadi Ibn Hashbal (14 km N Khamis Mushait), 18°27.34'N, 42°42.53'E, 1926 m, 2 Apr 2017, LT, D. Baiocchi leg., 1♀. Rijal Almaa, Wadi Kasan (3 km N Al Hubail), 18°06.981'N, 42°13.939'E, 451 m, 3 Apr 2017, LT, M.S. Abdel-Dayem and I. Rasool leg., 1♀ and 1♂; *ibidem*, 18°07.12'N, 42°13.55'E, 489 m, 5 Apr 2017, LT, D. Baiocchi leg., 5♀ and 1♂. **Jizan Province**: Al Darb, Wadi Reem, 17°52.551'N, 42°16.664'E, 136 m, 5 Apr 2017, LT, M.S. Abdel-Dayem leg., 2♀ and 2♂. **Riyadh Province**: Hotat Bani Tamim, Ibex Reserve Protected Area, (W Hotat Bani Tamim), 23°21.07'N, 46°21.36'E, 709 m, 11 Apr 2017, LT, D. Baiocchi leg., 5♀.

##### Distribution.

Africa: Egypt, Sudan. Asia: Israel, Oman, Saudi Arabia, Yemen. A polycentric Afro-syroeremic species.

##### Notes.

This species was previously reported in the provinces of Asir, Al Bahah, Jizan, and Makkah (Hölzel 1982). They were collected from sandy and rocky areas with *Acacia* woodlands and Barbary fig shrubs at elevations of 136‒1926 m in southwestern and central Saudi Arabia.

#### Neuroleon modestus

Taxon classificationAnimaliaNeuropteraMyrmeleontidae

(Navás, 1912)

BF8BE642-2C1E-5904-81C0-31A55AEC16DF

##### Material examined.

**Al Bahah Province**: Al Makhwah, Wadi Reyam (NE Al Makhwah), 19°50'28"N, 41°22'34"E, 473 m, 7 Apr 2019, LT, D. Baiocchi et al. leg., 1♀. **Asir Province**: Rijal Almaa, Wadi Kasan (2 km N Al Hubail), 18°07.12'N, 42°13.55'E, 489 m, 5 Apr 2017, LT, D. Baiocchi leg., 1♀.

##### Distribution.

Africa: Benin, Burkina Faso, Cabo Verde, Cote d’Ivoire, Mali. Asia: Saudi Arabia, Yemen. An Afrotropical species.

##### Biology.

*Neuroleon modestus* occurs in grass savannas and grassy vegetation in cultivated areas (Michel and Akoudjin 2012).

##### Notes.

This species was reported by Hölzel (1988) as *Neuroleon sociorum* Hölzel and Ohm in Asir Province. The listed female specimens were collected with light trap in sandy areas with *Acacia* woodlands at low elevations of 473‒489 m in southwestern Saudi Arabia.

#### Neuroleon pardalice

Taxon classificationAnimaliaNeuropteraMyrmeleontidae

(Banks, 1911) (Fig. 3F)

73258049-0B39-56C1-8284-1E7696050DB3

##### Material examined.

**Al Bahah Province**: Al Mekhwah, Shada Al-A’Ala Nature Reserve, 19°52.598'N, 41°18.672'E, 892 m, 3 Mar 2015, LT, M. Mostafa et al. leg., 1♀; *ibidem*, 19°51.762'N, 41°18.089'E, 1225 m, 21 Apr 2014, LT, Al Dhafer et al. leg., 1♂; *ibidem*, 19°50'51"N, 41°18'06"E, 1358 m, 9 Apr 2019, LT, D. Baiocchi et al. leg., 2♀.

##### Distribution.

Africa: Burkina Faso, Eritrea, Ethiopia, Nigeria, Sudan. Asia: Saudi Arabia, Yemen. An Afrotropical species.

##### Notes.

This species was previously reported in Al Bahah and Asir provinces (Hölzel 1982). The specimens were collected with light traps in sandy areas with *Acacia* woodlands at 892–1358 m in southwestern Saudi Arabia.

#### Neuroleon tenellus

Taxon classificationAnimaliaNeuropteraMyrmeleontidae

(Klug in Ehrenberg, 1834)

D9781F37-498C-5098-9F08-22600EF86D48

##### Material examined.

**Al Bahah Province**: Al Mekhwah, Shada Al-A’Ala Nature Reserve, 19°51.682'N, 41°18.263'E, 1291 m, 29 Mar 2017, LT, M.S. Abdel-Dayem leg., 1♂. **Asir Province**: Rijal Almaa, Wadi Kasan (3 km N Al Hubail), 18°06.981'N, 42°13.939'E, 451 m, 3 Apr 2017, LT, M.S. Abdel-Dayem and I. Rasool leg., 5♀ and 1♂; *ibidem*, 18°07.12'N, 42°13.55'E, 489 m, 5 Apr 2017, LT, D. Baiocchi leg., 17♀ and 1♂; *ibidem*, 1♀ and 1♂ (AL). **Jizan Province**: Al Darb, Wadi Reem, 17°52.551'N, 42°16.664'E, 136 m, 5 Apr 2017, LT, M.S. Abdel-Dayem leg., 1♂; *ibidem*, 17°51.56'N, 42°16.21'E, 139 m, 3 Apr 2017, LT, D. Baiocchi leg., 1♂. **Riyadh Province**: Riyadh, NW Al Uyaynah, 24°53.33'N, 46°17.40'E, 761 m, 10 Apr 2016, LT, D. Baiocchi leg., 2♀ and 1♂ (AL).

##### Distribution.

Africa: Algeria, Egypt, Eritrea, Ethiopia, Libya, Morocco, Mauritania, Sudan, Tunisia. Asia: Afghanistan, Azerbaijan, Cyprus, Israel, Iran, Iraq, Kyrgyzstan, Lebanon, Oman, Saudi Arabia, Turkey, Tajikistan, Turkmenistan, Uzbekistan. Europe: Greece, North Macedonia. A polycentric Afro-asianeremic species.

##### Biology.

*Neuroleon tenellus* is not well known and is usually documented in arid habitats. The larvae have not been documented (Aspöck et al. 1980; Badano et al. 2018).

##### Notes.

The species was previously documented in Eastern Province and Riyadh provinces (Hölzel 1982). The specimens were collected in *Acacia* woodlands on both rocky and sandy soils at elevations of 136‒1291 m in southwestern and central Saudi Arabia.

#### Neuroleon virgineus

Taxon classificationAnimaliaNeuropteraMyrmeleontidae

Hölzel, 1983

E21C77A5-FD1C-50D6-BF88-3F590B420BD1

##### Material examined.

**Al Bahah Province**: Al Makhwah, Wadi Reyam (NE Al Makhwah), 19°50'28"N, 41°22'34"E, 473 m, 7 Apr 2019, LT, D. Baiocchi et al. leg., 4♀ and 1♂; *ibidem*, 1♀ and 1♂ (AL).

##### Distribution.

Asia: An endemic species to Saudi Arabia.

##### Notes.

This study presents the first record of this species after the original description from Makka Province (Hölzel 1983). The listed specimens were collected from sandy areas with *Acacia* woodlands at low elevation of 473 m in southwestern Saudi Arabia.

#### Distoleon asiricus

Taxon classificationAnimaliaNeuropteraMyrmeleontidae

Hölzel, 1983

B0952673-7691-53BF-9809-399517557801

##### Material examined.

**Al Bahah Province**: Al Mekhwah, Shada Al-A’Ala Nature Reserve, 19°51.066’N, 41°18.037’E, 1325, 20 Apr 2014, LT, Al Dhafer et al. leg., 1♂. **Asir Province**: Abha, Garf Raydah Nature Reserve, 18°11.749'N, 42°23.345'E, 1614 m, 6 Jun 2014, LT, Al Dhafer et al. leg., 1♂; *ibidem*, 31 Jul 2015, LT, Al Dhafer et al. leg., 1♀; *ibidem*, 20 Oct 2014, LT, Al Dhafer et al. leg., 1♀; *ibidem*, 18°11.618'N, 42°23.420'E, 1772 m, 31 Jul 2015, LT, Al Dhafer et al. leg., 1♀; *ibidem*, 18°11.679'N, 42°23.691'E, 1851 m, 6 Jun 2014, LT, Al Dhafer et al. leg., 1♀ and 3♂; *ibidem*, 20 Oct 2014, LT, Al Dhafer et al. leg., 1♀; *ibidem*, 18°11.766'N, 42°24.315'E, 2285 m, 6 Jun 2014, LT, Al Dhafer et al. leg., 1♀; *ibidem*, 31 Jul 2015, LT, Al Dhafer et al. leg., 1♂; *ibidem*, 18°11.884'N, 42°24.435'E, 2387 m, 28 Apr 2014, LT, Al Dhafer et al. leg., 1♀; *ibidem*, 6 Jun 2014, LT, Al Dhafer et al. leg., 2♀ and 1♂; *ibidem*, 18°12.095'N, 42°24.536'E, 2578 m, 6 Jun 2014, LT, Al Dhafer et al. leg., 1♀; *ibidem*, 20 Oct 2014, LT, Al Dhafer et al. leg., 1♀; Wadi Marabah (WSW Abha), 18°10.293'N, 42°22.195'E, 1150 m, 1 Apr 2017, LT, M.S. Abdel-Dayem leg., 1♀; *ibidem*, 18°10.18'N, 42°22.12'E, 1197 m, 16 Apr 2016, LT, D. Baiocchi leg., 1♂; *ibidem*, 1♀ (AL). **Riyadh Province**: Hotat Bani Tamim, Ibex Reserve Protected Area, (W Hotat Bani Tamim), 23°21.07'N, 46°21.36'E, 709 m, 11 Apr 2017, LT, D. Baiocchi leg., 1♀ and 2♂.

##### Distribution.

Asia: Saudi Arabia, United Arab Emirates, Yemen. An endemic species to the Arabian Peninsula.

##### Biology.

The listed records present further localities in Saudi Arabia from where this species has been recently described.

##### Notes.

It was previously reported in Asir and Al Bahah provinces in southwestern Saudi Arabia (Hölzel 1983). The listed specimens were collected from rocky areas with *Acacia* woodlands, Barbary fig shrublands, and juniper forest at high elevations (1150‒2387 m) in southwestern Saudi Arabia, and in sandy areas with *Acacia* woodlands at an elevation of 709 m in the central regions of Saudi Arabia.

#### Distoleon laticollis

Taxon classificationAnimaliaNeuropteraMyrmeleontidae

(Navás, 1913)

E8EEB8DA-4433-5C98-AD49-D4EB641B16AA

##### Material examined.

**Al Bahah Province**: Al Mandaq, Wadi Tourabah (E of An Na’Amah), 20°11'01"N, 41°18'42"E, 1826 m, 6 Apr 2019, LT, D. Baiocchi et al. leg., 1♀. Al Mekhwah, Shada Al-A’Ala Nature Reserve, 19°52.598'N, 41°18.672'E, 892 m, 2 Mar 2015, LT, A. Mostafa leg., 1♂; *ibidem*, 19°50'51"N, 41°18'06"E, 1358 m, 9 Apr 2019, LT, D. Baiocchi et al. leg., 1♀. **Asir Province**: Abha, WSW of Abha, Garf Raydah Nature Reserve, 18°11.749'N, 42°23.345'E, 1614 m, 5 Nov 2015, LT, Al Dhafer et al. leg., 1♀; *ibidem*, 18°11.679'N, 42°23.691'E, 1851 m, 6 Jun 2014, LT, Al Dhafer et al. leg., 1♀. Wadi Marabah (WSW Abha), 18°10.293'N, 42°22.195'E, 1150 m, 1 Apr 2017, LT, M.S. Abdel-Dayem leg., 1♂; *ibidem*, 18°10.18'N, 42°22.12'E, 1197 m, 1 Apr 2017, LT, D. Baiocchi leg., 2♀ and 3♂; *ibidem*, 16 Apr 2016, LT, D. Baiocchi leg., 1♂; *ibidem*, 1♂ (AL). Rijal Almaa, Wadi Kasan (2 km N Al Hubail), 18°07.12'N, 42°13.55'E, 489 m, 5 Apr 2017, LT, D. Baiocchi leg., 1♂. **Riyadh Province**: Hotat Bani Tamim, Ibex Reserve Protected Area, (W Hotat Bani Tamim), 23°21.07'N, 46°21.36'E, 709 m, 11 Apr 2017, LT, D. Baiocchi leg., 1♀ and 1♂.

##### Distribution.

Africa: Ethiopia, Sudan. Asia: Cyprus, Israel, Lebanon, Oman, Saudi Arabia, Syria, Turkey. A polycentric Afro-syroeremic species.

##### Biology.

Mainly unknown, associated with arid environments (Badano et al. 2018).

##### Notes.

This species was previously recorded in the mountains of southwestern Saudi Arabia, from Al Bahah and Makkah provinces (Hölzel 1982). The listed specimens were collected in *Acacia* woodlands in rocky and sandy soils and in rocky areas with Barbary fig shrub communities at different elevations (489‒1851 m) in southwestern and central Saudi Arabia.

#### Nemoleon secundus

Taxon classificationAnimaliaNeuropteraMyrmeleontidae

(Hölzel, 2002)

06527BD4-5963-501E-BBBC-76975765D889

##### Material examined.

**Al Bahah Province**: Al Mekhwah, Shada Al Asfal, Al-Hamadah, 20°10.750'N, 41°19.072'E, 554 m, 30 Mar 2017, LT, M.S. Abdel-Dayem leg., 5♀ and 1♂; *ibidem*, 1♀ and 1♂ (AL); Wadi Reyam (NE Al Makhwah), 19°50'28"N, 41°22'34"E, 473 m, 7 Apr 2019, LT, D. Baiocchi et al. leg., 1♀ and 4♂.

##### Distribution.

Asia: Oman, Yemen. An endemic species to the Arabian Peninsula.

##### Notes.

This study presents the first report in Saudi Arabia, with this species recently being described from Oman and Yemen (Hölzel, 2002). The listed specimens were collected from foothill *Acacia* woodlands at elevations of 473‒554 m in southwestern Saudi Arabia.

#### Pseudoformicaleo gracilis

Taxon classificationAnimaliaNeuropteraMyrmeleontidae

(Klug in Ehrenberg, 1834)

F1805D7A-7F44-5947-8640-470FF97B8E8C

##### Material examined.

**Asir Province**: Abha, Wadi Marabah (WSW Abha), 18°10.18'N, 42°22.12'E, 1197 m, 16 Apr 2016, LT, D. Baiocchi leg., 1ex. (AL).

##### Distribution.

Africa: Algeria, Egypt, Libya, Morocco, Tunisia. Asia: Iran, Israel, Lebanon, Oman, Russia, Saudi Arabia, Syria, Turkey, United Arab Emirates, Yemen. A polycentric Afro-syro-iranoeremic species.

##### Notes.

This species was previously recorded in east and southwest Saudi Arabia, in Eastern Province and Makkah provinces, respectively (Hölzel 1982). The listed specimen was collected from mountainous *Acacia* woodlands at an elevation of 1197 m in southwestern Saudi Arabia.

#### Creoleon elegans

Taxon classificationAnimaliaNeuropteraMyrmeleontidae

Hölzel, 1968

1A448479-F7EA-5A3D-A900-0B304152147A

##### Material examined.

**Asir Province**: Abha, Wadi Marabah (WSW Abha), 18°10.18'N, 42°22.12'E, 1197 m, 1 Apr 2017, LT, D. Baiocchi leg., 2♀.

##### Distribution.

Asia: Israel, Iran, Iraq, Pakistan, Saudi Arabia, Syria. Syro-iranoeremic species.

##### Biology.

The larvae possibly inhabit sandy soils, like congeners.

##### Notes.

This species was previously reported in central (Riyadh Province) and southwestern (Makkah Province) Saudi Arabia (Hölzel 1982). The listed specimens were collected from rocky areas with *Acacia* woodlands at an elevation of 1197 m in the highlands of southwestern Saudi Arabia.

#### Creoleon griseus

Taxon classificationAnimaliaNeuropteraMyrmeleontidae

(Klug in Ehrenberg, 1834)

51E3D986-BEC9-541A-A444-0251A8AFE149

##### Material examined.

**Asir Province**: Khamis Mushait, Wadi Ibn Hashbal (14 km N Khamis Mushait), 18°27.34'N, 42°42.53'E, 1926 m, 2 Apr 2017, LT, D. Baiocchi leg., 1♂.

##### Distribution.

Africa: Egypt, Senegal, Sudan, Tunisia. Asia: Afghanistan, India, Iran, Iraq, Israel, Oman, Pakistan, Saudi Arabia, Syria, Yemen. Europe: Spain. A widespread polycentric Afro-syro-iranoeremic species.

##### Biology.

The larva possibly inhabits sandy soils, like congeners.

##### Notes.

It was previously recorded in central Saudi Arabia, in Riyadh Province (Hölzel 1982). The listed male specimen was collected from sandy areas with *Acacia* woodlands at an elevation of 192 m in southwestern Saudi Arabia.

#### Creoleon persicus

Taxon classificationAnimaliaNeuropteraMyrmeleontidae

Hölzel, 1972

2BD051EA-9914-5141-ACC0-1E623A773ECA

##### Material examined.

**Al Bahah Province**: Al Mekhwah, Shada Al Asfal, Al-Hamadah, 20°10.750'N, 41°19.072'E, 554 m, 30 Mar 2017, LT, M.S. Abdel-Dayem leg., 1♀. **Asir Province**: Khamis Mushait, Wadi Ibn Hashbal (14 km N Khamis Mushait), 18°27.34'N, 42°42.53'E, 1926 m, 2 Apr 2017, LT, D. Baiocchi leg., 1♀.

##### Distribution.

Asia: Afghanistan, Iran, Israel, Saudi Arabia. A Syro-iranoeremic species.

##### Biology.

The larva possibly inhabits sandy soils, like congeners.

##### Notes.

It was documented in northern and southwestern Saudi Arabia, in Tabouk, Asir and Riyadh provinces (Hölzel 1982). The listed specimens were attracted to light traps in sandy areas with *Acacia* woodlands at elevations between 554‒1926 m in southwestern Saudi Arabia.

#### Creoleon ultimus

Taxon classificationAnimaliaNeuropteraMyrmeleontidae

Hölzel, 1983

60F0B9B3-F436-5C1F-9A4E-76E67B07EFC4

##### Material examined.

**Al Bahah Province**: Al Mekhwah, Shada Al-A’Ala Nature Reserve, 19°50'51"N, 41°18'06"E, 1358 m, 9 Apr 2019, LT, D. Baiocchi et al. leg., 1♀ and 1♂. **Asir Province**: Khamis Mushait, Wadi Ibn Hashbal (14 km N Khamis Mushait), 18°27.558'N, 42°42.876'E, 1926 m, 2 Apr 2017, LT, M.S. Abdel-Dayem leg., 1♂.

##### Distribution.

Asia: An endemic species to Saudi Arabia.

##### Biology.

The larva possibly inhabits sandy soils, like congeners.

##### Notes.

This study presents the first report of this species after the original description, based on one male and one female collected in Al Bahah Province in southwestern Saudi Arabia (Hölzel 1983). The listed male specimens were collected in sandy and rocky areas with *Acacia* woodlands at elevations of 1358‒1926 m in the highlands of southwestern Saudi Arabia.

### Ascalaphidae Lefèbvre, 1842

#### Ascalaphus festivus

Taxon classificationAnimaliaNeuropteraAscalaphidae

(Rambur, 1842)

1A54A3CE-10E2-537B-9BAE-D4D06E141B06

##### Material examined.

**Al Bahah Province**: Al Mandaq, Wadi Turubah, 20°14.369'N, 41°15.234'E, 1757 m, 3 Jun 2012, H. Al Dhafer et al. leg., 3♀. **Asir Province**: Al Magardah, Wadi Al Talalie, 18°59.840'N, 41°43.910'E, 242 m, 1 Jun 2012, B. Kondratiff and H. Al Dahfer leg., SW, 1♀. Rijal Almaa, Wadi Kasan (3 km N Al Hubail), 18°06.981'N, 42°13.939'E, 451 m, 3 Apr 2017, LT, M.S. Abdel-Dayem and I. Rasool leg., 1♂; *ibidem*, 18°06'57"N, 42°13'55"E, 462 m, 12 Apr 2019, LT, D. Baiocchi et al. leg., 1♀ (AL); *ibidem*, 18°07.12'N, 42°13.55'E, 489 m, 5 Apr 2017, LT, D. Baiocchi leg., 3♂. **Jizan Province**: Al Darb, Wadi Reem, 17°52.551'N, 42°16.664'E, 136 m, 5 Apr 2017, LT, M.S. Abdel-Dayem leg., 1♀.

##### Distribution.

Africa: widespread, including Cabo Verde and Madagascar. Asia: Israel, Oman, Saudi Arabia, United Arab Emirates, Yemen. Europe: Italy. An Afrotropical species.

##### Biology.

It is commonly collected with light traps in open savannah areas that are sometimes cultivated or grazed by cattle (Tjeder 1980).

##### Notes.

This species was previously reported in Eastern Province, Jizan, and Riyadh provinces (Hölzel 1983). The specimens were collected in sandy areas with *Acacia* woodlands at elevations of 136‒1757 m in southwestern Saudi Arabia.

#### Stylascalaphus krueperi

Taxon classificationAnimaliaNeuropteraAscalaphidae

(van der Weele, 1909) (Fig. 3G)

64EDAFCA-872C-5DC5-86DC-3F46903901FF

##### Material examined.

**Al Bahah Province**: Al Mekhwah, Shada Al-A’Ala Nature Reserve, 19°52.598'N, 41°18.672'E, 892 m, 24 Apr 2014, LT, Al Dhafer et al. leg., 1♀; *ibidem*, 23 Aug 2014, LT, Al Dhafer et al. leg., 1♀.

##### Distribution.

Africa: Algeria, Morocco (Ábrahám 2017), Egypt. Asia: Jordan, Syria. An Afro-syroeremic species.

##### Notes.

This species is a new listing for fauna in the Arabian Peninsula. The listed female specimens were collected in sandy areas with *Acacia* woodlands at an elevation of 892 m in southwestern Saudi Arabia.

#### Aspoeckiella gallagheri

Taxon classificationAnimaliaNeuropteraAscalaphidae

Hölzel, 2004 (Fig. 3H)

DF45730E-A1D9-566B-A7C1-7FA1364033E5

##### Material examined.

**Al Bahah Province**: Al Mekhwah, Shada Al-A’Ala Nature Reserve, 19°57.686'N, 41°18.262'E, 607 m, 9 Apr 2019, LT, D. Baiocchi leg., 1♀. **Jizan Province**: Al Darb, Wadi Reem, 17°51.56'N, 42°16.21'E, 139 m, 3 Apr 2017, LT, D. Baiocchi leg., 1♂.

##### Distribution.

Asia: Oman, United Arab Emirates. An endemic species to the Arabian Peninsula.

##### Notes.

This study presents the first report of this species in Saudi Arabia, which was originally described from Oman and the United Arab Emirates (Hölzel 2004). The listed specimens were collected at low elevations (139‒607 m) in sandy areas with *Acacia* woodlands in southwestern Saudi Arabia.

#### Bubopsis hamata

Taxon classificationAnimaliaNeuropteraAscalaphidae

(Klug in Ehrenberg, 1834)

81400D88-00A2-56D6-AE8D-0072A0ADABDE

##### Material examined.

**Al Bahah Province**: Al Mekhwah, Shada Al-A’Ala Nature Reserve, 19°50.51'N, 41°18.06'E, 1358 m, 14 Apr 2016, LT, D. Baiocchi leg., 1♀. **Riyadh Province**: Hotat Bani Tamim, Ibex Reserve Protected Area (W Hotat Bani Tamim), 23°21.07'N, 46°21.36'E, 709 m, 11 Apr 2017, LT, D. Baiocchi leg., 2♂ (AL). Riyadh, Wadi Hanifa, 24°54.422'N, 46°10.903'E, 809 m, LT, 22 Apr 2017, M. Abdel-Dayem et al. leg., 1♀ and 2♂.

##### Distribution.

Africa: Egypt. Asia: Azerbaijan, Georgia, Iran, Iraq, Israel, Jordan, Kyrgyzstan, Lebanon, Saudi Arabia, Syria, Turkey, Turkmenistan, United Arab Emirates. Europe: Greece. An Asianeremic species.

##### Biology.

It is frequently collected by light traps; adults tend to inhabit steppe-like habitats, and rocky grasslands with long stalks (Dobosz and Ábrahám 2007).

##### Notes.

The species was previously reported in Asir, Madinah, and Riyadh provinces (Hölzel 1983). The listed specimens were collected with light traps in mountainous areas and sandy areas with *Acacia* woodlands at elevations of 709‒1358 m in southwestern and central Saudi Arabia, respectively.

#### Tmesibasis larseni

Taxon classificationAnimaliaNeuropteraAscalaphidae

Hölzel, 1983

CD331CEF-CEAD-5C81-A074-844997E0008E

##### Material examined.

**Al Bahah Province**: Al Mekhwah, Shada Al-A’Ala Nature, 19°51.066'N, 41°18.037'E, 1325 m, 24 Feb 2014, Al Dhafer et al. leg., 1♂; *ibidem*, 19°50.51'N, 41°18.06'E, 1358 m, 14 Apr 2016, LT, D. Baiocchi leg., 1♀ (AL). **Asir Province**: Abha, WSW of Abha, Garf Raydah Nature Reserve, 18°11.749'N, 42°23.345'E, 1614 m, 24 Mar 2014, LT, S.A. El-Sonbati leg., 1♀; *ibidem*, 5 Sep 2015, LT, Al Dhafer et al. leg., 1♀; Wadi Marabah (WSW Abha), 18°10.18'N, 42°22.12'E, 1197 m, 16 Apr 2016, LT, D. Baiocchi leg., 1♂ (AL). Rijal Almaa, Wadi Kasan (2 km N Al Hubail), 18°07.12'N, 42°13.55'E, 489 m, 5 Apr 2017, LT, D. Baiocchi leg., 1♀. **Jizan Province**: Al Darb, Wadi Reem, 17°52.551'N, 42°16.664'E, 136 m, 5 Apr 2017, LT, M.S. Abdel-Dayem leg., 1♀.

##### Distribution.

Asia: Oman, Saudi Arabia, Yemen. An endemic species to the Arabian Peninsula.

##### Notes.

The listed records for this species extend existing published Saudi Arabian localities from Gizan, based on a single female specimen (Hölzel 1983). The listed specimens were collected in rocky and sandy areas with *Acacia* woodlands and rocky areas with Barbary fig shrub communities at elevations of 136‒1358 m in southwestern Saudi Arabia.

## Discussion

Between 2014 and 2019, specimens of 61 lacewing species belonging to seven families were collected in Saudi Arabia. Also, two species belonging to *Dielocroce* and *Pseudomallada* were identified only to genus level. The families included are: Ascalaphidae (5 species), Berothidae (3 species), Chrysopidae (10 species), Hemerobiidae (1 species), Mantispidae (2 species), Myrmeleontidae (36 species), and Nemopteridae (6 species). This list includes six new species records for the country. Of these, three species are new records to the Arabian Peninsula: *Mantispa aphavexelte* Aspöck & Aspöck, *Omoleon jeanneli* Navás, and *Stylascalaphus krueperi* (van der Weele). The other three are new records to Saudi Arabia only: *Aspoeckiella gallagheri* Hölzel, *Bankisus maculosus* Hölzel and *Nemoleon secundus* (Hölzel). Notably, *Mantispa aphavexelte* was recorded close to the southern boundary of its distributional range. This study also provides the first record for eight species since their original description: *Creoleon ultimus* Hölzel, *Cueta amseli* Hölzel, *C. asirica* Hölzel, *Distoleon asiricus* Hölzel, *Geyria pallida* Hölzel, *Neuroleon delicatus* Hölzel, *Neuroleon virgineus* Hölzel, and *Solter buettikeri* Hölzel.

**Figure 2.**
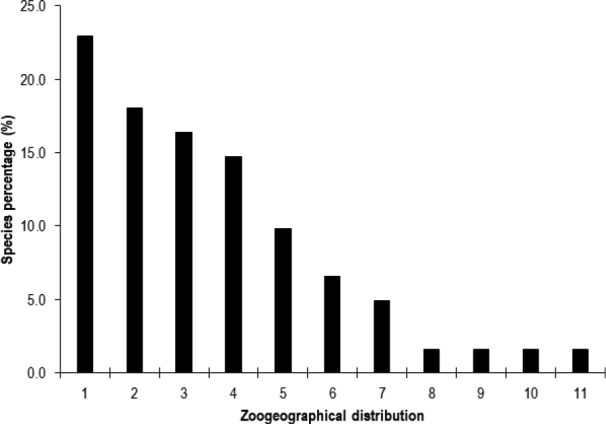
Zoogeographical composition of lacewing fauna in Saudi Arabia **1** afro-syroeremic **2** afrotropical **3** Arabian Endemic **4** afro-syro-iranoeremic **5** Saudi Endemic **6** syro-iranoeremic **7** palaearctic **8** afro-asianeremic **9** asianeremic **10** Palaearctic-Afrotropical-Oriental **11** syroeremic.

Many of the lacewing species documented in this study are characteristic of fauna from eremial bioregions (47.5%; Fig. 2), particularly Afro-syroeremic species (14 spp., 23.0%), followed by Afro-syro-iranoeremic species (9 spp., 14.8) and Afrotropical species (11 spp., 18.0%). Three species were representatives of Palaearctic species (4.9%): *Cueta lineosa* (Rambur), *Mantispa aphavexelte* Aspöck & Aspöck and *Pseudomallada venosus* (Rambur). *Chrysoperla carnea* (Stephens) was the only species that had a wide distributional range (Afrotropical, Oriental and Palaearctic regions). These findings confirm that the lacewing species recorded in Saudi Arabia have strong relationships with the eremic fauna of North Africa (Afroeremic) and Asia (Syroeremic, Iranoeremic, and Turanoeremic), as well as the Afrotropical fauna. The high percentage of eremic (Saharo-Arabian and Saharo-Sindian) components in the fauna of Saudi Arabia has been previously documented (Larsen 1984; Penati and Vienan 2006; Abel-Dayem et al. 2017, 2018, 2019).

A large number of species are endemic to Saudi Arabia and the Arabian Peninsula (16 spp., 26.2%). *Creoleon ultimus* Hölzel, *Cueta asirica* Hölzel, *Neuroleon delicatus* Hölzel, *N. virgineus* Hölzel, *Podallea arabica* Aspöck & Aspöck, and *Solter buettikeri* Hölzel, are known as endemic to Saudi Arabia. Ten species are endemic to the Arabian Peninsula, being distributed in Oman, Saudi Arabia, the United Arab Emirates, and Yemen: *Aspoeckiella gallagheri* Hölzel, *Bankisus maculosus* Hölzel, *Centroclisis speciosa* Hölzel, *Cueta amseli* Hölzel, *Distoleon asiricus* Hölzel, *Geyria pallida* Hölzel, *Iranoleon arabicus* Hölzel, *Nemoleon secundus* (Hölzel), *Pseudomallada arabicus* (Hölzel), and *Tmesibasis larseni* Hölzel. The southwestern region of Saudi Arabia is particularly rich in insect species (Larsen 1984; Abuzinada et al. 2001; Ziani et al. 2019), reflecting the high number of endemic lacewing species that are mostly syroeremic species (Hölzel 1998). A high percentage of endemic species to Saudi Arabia are emerging partly because current knowledge on Afrotropical and Oriental fauna is limited and partly because the Arabian Peninsula is in the transitional zone between Palaearctic and Afrotropical regions, as well as being close to Oriental regions.

**Figure 3.**
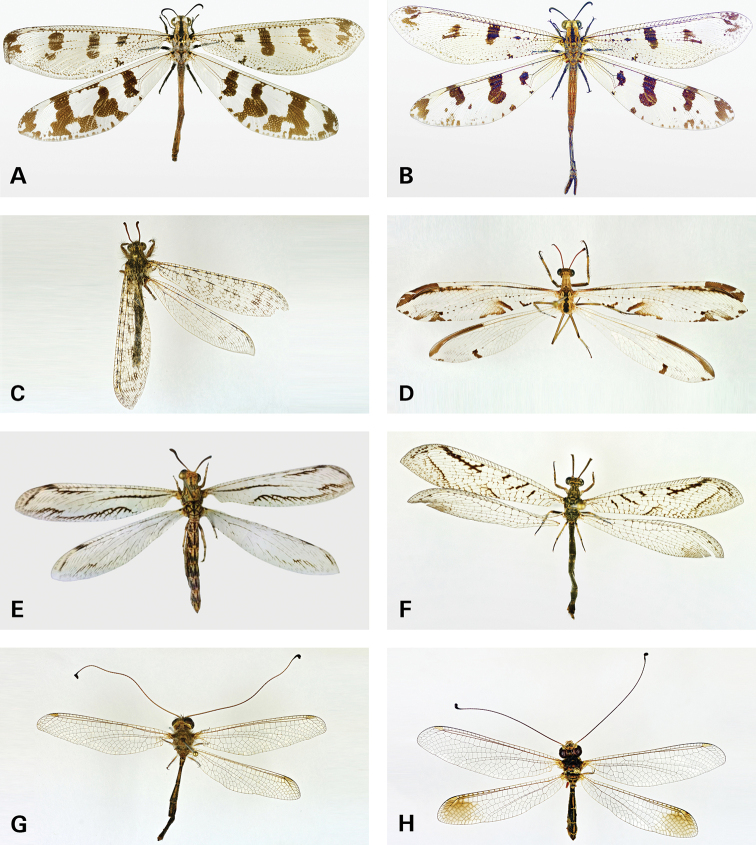
Habitus of lacewing species in Saudi Arabia **A***Goniocercus walkeri***B***Stenares irroratus***C***Centroclisis speciosa***D***Omoleon jeanneli***E***Gepus invisus***F***Neuroleon pardalice***G***Stylascalaphus krueperi***H***Aspoeckiella gallagheri*.

According to Oswald (2019), 170 lacewing taxa are known from Saudi Arabia in addition to six new country records from our study (Appendix I). We listed 61 species of the (now) 176 taxa known from Saudi Arabia, so we sampled and reported a little more than one third (34.7%) of the total known Neuropterida fauna of Saudi Arabia. The most prominent in our survey, is the absence of Coniopterygidae (Appendix I) which are very hardly to detect (a very small insects) without specific researches. The huge differences of knowledge of this family in Yemen (57 spp.) and Saudi Arabia (10 spp.) is due only to the lack of a specific research of Coniopterygidae in Saudi Arabia.

Despite a large number of studies existing on the lacewing fauna of the Arabian Peninsula (Meinander 1979, 1980; Hölzel 1980, 1982, 1983, 1988, 1995, 1998; Sziráki and van Harten 2006; Sziráki 2010), including the current study, there is still a paucity of knowledge about the lacewing fauna in the Arabian Peninsula. This issue is reflected by the rate of discovery of lacewing species in the Arabian Peninsula has not yet reached a plateau. The addition of lacewing species after more thorough sampling efforts is expected to provide more reliable biogeographical patterns on this group. Further studies on the lacewing fauna of Saudi Arabia should also focus on the biology and ecology of this group.

## Supplementary Material

XML Treatment for Italochrysa bimaculata

XML Treatment for Pseudomallada amseli

XML Treatment for Pseudomallada arabicus

XML Treatment for Pseudomallada spadix

XML Treatment for Pseudomallada venosus

XML Treatment for Pseudomallada

XML Treatment for Chrysoperla carnea

XML Treatment for Brinckochrysa alfierii

XML Treatment for Brinckochrysa chlorosoma

XML Treatment for Chrysemosa andresi

XML Treatment for Micromus sjostedti

XML Treatment for Afromantispa nana

XML Treatment for Mantispa aphavexelte

XML Treatment for Nodalla eatoni

XML Treatment for Nodalla saharica

XML Treatment for Podallea arabica

XML Treatment for Croce aristata

XML Treatment for Dielocroce berlandi

XML Treatment for Dielocroce chobauti

XML Treatment for Dielocroce elegans

XML Treatment for Dielocroce

XML Treatment for Halter halteratus

XML Treatment for Goniocercus walkeri

XML Treatment for Stenares irroratus

XML Treatment for Fadrina formosa

XML Treatment for Centroclisis speciosa

XML Treatment for Myrmecaelurus lepidus

XML Treatment for Iranoleon arabicus

XML Treatment for Lopezus fedtschenkoi

XML Treatment for Gepus invisus

XML Treatment for Solter buettikeri

XML Treatment for Solter propheticus

XML Treatment for Cueta amseli

XML Treatment for Cueta asirica

XML Treatment for Cueta lineosa

XML Treatment for Cueta pallens

XML Treatment for Myrmeleon caliginosus

XML Treatment for Myrmeleon fasciatus

XML Treatment for Bankisus maculosus

XML Treatment for Omoleon jeanneli

XML Treatment for Geyria lepidula

XML Treatment for Geyria pallida

XML Treatment for Neuroleon asirensis

XML Treatment for Neuroleon delicatus

XML Treatment for Neuroleon leptaleus

XML Treatment for Neuroleon lugubris

XML Treatment for Neuroleon modestus

XML Treatment for Neuroleon pardalice

XML Treatment for Neuroleon tenellus

XML Treatment for Neuroleon virgineus

XML Treatment for Distoleon asiricus

XML Treatment for Distoleon laticollis

XML Treatment for Nemoleon secundus

XML Treatment for Pseudoformicaleo gracilis

XML Treatment for Creoleon elegans

XML Treatment for Creoleon griseus

XML Treatment for Creoleon persicus

XML Treatment for Creoleon ultimus

XML Treatment for Ascalaphus festivus

XML Treatment for Stylascalaphus krueperi

XML Treatment for Aspoeckiella gallagheri

XML Treatment for Bubopsis hamata

XML Treatment for Tmesibasis larseni

## Appendix I

**Table:** List of known lacewings from Saudi Arabia.

Family	Species	This study
Ascalaphidae	*Ascalaphus dicax* Walker, 1853	
Ascalaphidae	*Ascalaphus festivus* (Rambur, 1842)	+
Ascalaphidae	*Aspoeckiella gallagheri* Hölzel, 2004	+
Ascalaphidae	*Bubopsis hamata* (Klug in Ehrenberg, 1834)	+
Ascalaphidae	*Stylascalaphus krueperi* (van der Weele, 1909)	+
Ascalaphidae	*Tmesibasis larseni* Hölzel, 1983	+
Berothidae	*Nodalla eatoni* (McLachlan, 1898)	+
Berothidae	*Nodalla saharica* (Esben-Petersen, 1920)	+
Berothidae	*Podallea arabica* Aspöck & Aspöck, 1981	+
Chrysopidae	*Brinckochrysa alfierii* (Navás, 1926)	+
Chrysopidae	*Brinckochrysa chlorosoma* (Navás, 1914)	+
Chrysopidae	*Brinckochrysa plagata* (Navás, 1929)	
Chrysopidae	*Chrysemosa andresi* (Navás, 1915)	+
Chrysopidae	*Chrysemosa mosconica* (Navás, 1931)	
Chrysopidae	*Chrysemosa sodomensis* (Hölzel, 1982)	
Chrysopidae	*Chrysopa sogdianica* McLachlan in Fedchenko, 1875	
Chrysopidae	*Chrysoperla carnea* (Stephens, 1836)	+
Chrysopidae	*Chrysoperla mutata* (McLachlan, 1898)	
Chrysopidae	*Italochrysa asirensis* Hölzel, 1980	
Chrysopidae	*Italochrysa bimaculata* Hölzel, 1980	+
Chrysopidae	*Italochrysa pittawayi* Hölzel, 1988	
Chrysopidae	*Italochrysa stigmatica* (Rambur, 1838)	
Chrysopidae	*Pseudomallada amseli* (Hölzel, 1980)	+
Chrysopidae	*Pseudomallada arabicus* (Hölzel, 1995)	+
Chrysopidae	*Pseudomallada healdi* (Navás, 1926)	
Chrysopidae	*Pseudomallada nicolainus* (Navás, 1929)	
Chrysopidae	*Pseudomallada phlebius* (Navas, 1927)	
Chrysopidae	*Pseudomallada spadix* (Hölzel, 1988)	+
Chrysopidae	*Pseudomallada venosus* (Rambur, 1838)	+
Chrysopidae	*Suarius alisteri* (Navás, 1914)	
Chrysopidae	*Suarius caviceps* (McLachlan, 1898)	
Chrysopidae	*Suarius gobiensis* (Tjeder, 1936)	
Chrysopidae	*Suarius mongolica* (Tjeder, 1936)	
Chrysopidae	*Suarius walsinghami walsinghami* Navás, 1914	
Coniopterygidae	*Aleuropteryx arabica* Meinander, 1977	
Coniopterygidae	*Aleuropteryx vartianorum* Aspöck & Aspöck, 1967	
Coniopterygidae	*Coniopteryx deserta* Meinander, 1979	
Coniopterygidae	*Coniopteryx mucrogonarcuata* Meinander, 1979	
Coniopterygidae	*Coniopteryx ressli* Rausch & Aspöck, 1978	
Coniopterygidae	*Coniopteryx venustula* Rausch & Aspöck, 1978	
Coniopterygidae	*Coniopteryx wittmeri* Meinander, 1979	
Coniopterygidae	*Cryptoscenea serrata* (Meinander, 1979)	
Coniopterygidae	*Hemisemidalis pallida* (Withycombe, 1924)	
Coniopterygidae	*Nimboa macroptera* Aspöck & Aspöck, 1965	
Hemerobiidae	*Hemerobius reconditus* Navás, 1914	
Hemerobiidae	*Micromus sjostedti* van der Weele, 1910	+
Hemerobiidae	*Sympherobius fallax* Navás, 1908	
Hemerobiidae	*Wesmaelius navasi* (Andréu, 1911)	
Hemerobiidae	*Wesmaelius saudiarabicus* Hölzel, 1988	
Mantispidae	*Afromantispa nana* (Erichson, 1839)	+
Mantispidae	*Mantispa aphavexelte* Aspöck & Aspöck, 1994	+
Mantispidae	*Mantispa scabricollis* McLachlan in Fedchenko, 1875	
Myrmeleontidae	*Acanthaclisis mesopotamica* Hölzel, 1972	
Myrmeleontidae	*Bankisus maculosus* Hölzel, 1983	+
Myrmeleontidae	*Centroclisis cervina* (Gerstaecker, 1863)	
Myrmeleontidae	*Centroclisis distincta* (Rambur, 1842)	
Myrmeleontidae	*Centroclisis speciosa* Hölzel, 1983	+
Myrmeleontidae	*Creoleon cervinus* Hölzel, 1983	
Myrmeleontidae	*Creoleon cinerascens* (Navás, 1912)	
Myrmeleontidae	*Creoleon desertus* Hölzel, 1982	
Myrmeleontidae	*Creoleon elegans* Hölzel, 1968	+
Myrmeleontidae	*Creoleon griseus* (Klug in Ehrenberg, 1834)	+
Myrmeleontidae	*Creoleon neftanus* Navás, 1930	
Myrmeleontidae	*Creoleon neurasthenicus* (Navás, 1913)	
Myrmeleontidae	*Creoleon parvulus* Hölzel, 1983	
Myrmeleontidae	*Creoleon persicus* Hölzel, 1972	+
Myrmeleontidae	*Creoleon pullus* Hölzel, 1983	
Myrmeleontidae	*Creoleon ultimus* Hölzel, 1983	+
Myrmeleontidae	*Cueta amseli* Hölzel, 1982	+
Myrmeleontidae	*Cueta asirica* Hölzel, 1982	+
Myrmeleontidae	*Cueta clara* Hölzel, 1981	
Myrmeleontidae	*Cueta divisa* (Navás, 1912)	
Myrmeleontidae	*Cueta genialis* Hölzel, 1988	
Myrmeleontidae	*Cueta lineosa* (Rambur, 1842)	+
Myrmeleontidae	*Cueta modesta* Hölzel, 1972	
Myrmeleontidae	*Cueta pallens* (Klug in Ehrenberg, 1834)	+
Myrmeleontidae	*Cueta paula* Hölzel, 1983	
Myrmeleontidae	*Cueta pusilla* Hölzel, 1983	
Myrmeleontidae	*Cueta striata* Kimmins, 1943	
Myrmeleontidae	*Cueta virgata* (Klug in Ehrenberg, 1834)	
Myrmeleontidae	*Distoleon asiricus* Hölzel, 1983	+
Myrmeleontidae	*Distoleon laticollis* (Navás, 1913)	+
Myrmeleontidae	*Fadrina formosa* (Hölzel, 1981)	+
Myrmeleontidae	*Ganguilus flavipennis* (Navás, 1932)	
Myrmeleontidae	*Ganguilus oblitus* (Navás, 1914)	
Myrmeleontidae	*Ganguilus pallescens* Navás, 1912	
Myrmeleontidae	*Ganguilus pulchellus* (Banks, 1911)	
Myrmeleontidae	*Gepella modesta* Hölzel, 1968	
Myrmeleontidae	*Gepus cunctatus* Hölzel, 1982	
Myrmeleontidae	*Gepus invisus* Navás, 1912	+
Myrmeleontidae	*Geyria arabica* Hölzel, 1983	
Myrmeleontidae	*Geyria lepidula* (Navás, 1912)	+
Myrmeleontidae	*Geyria pallida* Hölzel, 1983	+
Myrmeleontidae	*Geyria saharica* Esben-Petersen, 1920	
Myrmeleontidae	*Goniocercus klugi* (Kolbe, 1898)	
Myrmeleontidae	*Goniocercus walkeri* (McLachlan, 1894)	+
Myrmeleontidae	*Iranoleon arabicus* Hölzel, 1982	+
Myrmeleontidae	*Iranoleon darius* Hölzel, 1972	
Myrmeleontidae	*Isoleon arabicus* Hölzel, 1972	
Myrmeleontidae	*Lopezus arabicus* Hölzel, 1972	
Myrmeleontidae	*Lopezus fedtschenkoi* (McLachlan in Fedchenko, 1875)	+
Myrmeleontidae	*Macronemurus delicatulus* Morton, 1926	
Myrmeleontidae	*Mesonemurus harterti* Navás, 1919	
Myrmeleontidae	*Myrmecaelurus acerbus* (Walker, 1853)	
Myrmeleontidae	*Myrmecaelurus laetus* (Klug in Ehrenberg, 1834)	
Myrmeleontidae	*Myrmecaelurus lepidus* (Klug in Ehrenberg, 1834)	+
Myrmeleontidae	*Myrmecaelurus lobatus* Navás, 1912	
Myrmeleontidae	*Myrmecaelurus luridus* Hölzel, 1983	
Myrmeleontidae	*Myrmecaelurus obscurus* Hölzel, 1983	
Myrmeleontidae	*Myrmecaelurus parvulus* Hölzel, 1982	
Myrmeleontidae	*Myrmecaelurus peterseni* Kimmins, 1943	
Myrmeleontidae	*Myrmecaelurus philbyi* Kimmins, 1943	
Myrmeleontidae	*Myrmecaelurus pittawayi* Hölzel, 1983	
Myrmeleontidae	*Myrmecaelurus saudiarabicus* Hölzel, 1982	
Myrmeleontidae	*Myrmeleon alternans* Brullé in Webb & Berthelot, 1839	
Myrmeleontidae	*Myrmeleon caliginosus* Hölzel & Ohm, 1983	+
Myrmeleontidae	*Myrmeleon fasciatus* (Navás, 1912)	+
Myrmeleontidae	*Myrmeleon hyalinus* Olivier, 1811	
Myrmeleontidae	*Myrmeleon pellucidus* Hölzel, 1988	
Myrmeleontidae	*Myrmeleon vittatus* Olivier, 1811	
Myrmeleontidae	*Myrmeleon wismanni* (Navás, 1936)	
Myrmeleontidae	*Naya palpalis* (Klapálek, 1914)	
Myrmeleontidae	*Nemoleon secundus* (Hölzel, 2002)	+
Myrmeleontidae	*Neuroleon amseli* Hölzel, 1983	
Myrmeleontidae	*Neuroleon asirensis* Hölzel, 1983	+
Myrmeleontidae	*Neuroleon delicatus* Hölzel, 1983	+
Myrmeleontidae	*Neuroleon erato* Hölzel, 1972	
Myrmeleontidae	*Neuroleon gracilis* (Navás, 1926)	
Myrmeleontidae	*Neuroleon leptaleus* (Navás, 1912)	+
Myrmeleontidae	*Neuroleon lugubris* (Navás, 1926)	+
Myrmeleontidae	*Neuroleon modestus* (Navás, 1912)	+
Myrmeleontidae	*Neuroleon nubilatus* (Navás, 1912)	
Myrmeleontidae	*Neuroleon pardalice* (Banks, 1911)	+
Myrmeleontidae	*Neuroleon parvus* Kimmins, 1943	
Myrmeleontidae	*Neuroleon taifensis* Kimmins, 1943	
Myrmeleontidae	*Neuroleon tenellus* (Klug in Ehrenberg, 1834)	+
Myrmeleontidae	*Neuroleon virgineus* Hölzel, 1983	+
Myrmeleontidae	*Noaleon limbatellus* (Navás, 1913)	
Myrmeleontidae	*Nophis flava* Hölzel, 1972	
Myrmeleontidae	*Nophis luteus* Hölzel, 1972	
Myrmeleontidae	*Nophis teillardi* Navás, 1912	
Myrmeleontidae	*Omoleon jeanneli* Navás, 1936	+
Myrmeleontidae	*Palpares angustus* McLachlan, 1898	
Myrmeleontidae	*Palpares cephalotes* (Klug in Ehrenberg, 1834)	
Myrmeleontidae	*Palpares papilionoides* (Klug in Ehrenberg, 1834)	
Myrmeleontidae	*Palpares venustus* Hölzel, 1988	
Myrmeleontidae	*Parapalpares dispar* (Navás, 1912)	
Myrmeleontidae	*Phanoclisis longicollis* (Rambur, 1842)	
Myrmeleontidae	*Pseudoformicaleo gracilis* (Klug in Ehrenberg, 1834)	+
Myrmeleontidae	*Quinemurus cinereus* Kimmins, 1943	
Myrmeleontidae	*Solter buettikeri* Hölzel, 1982	+
Myrmeleontidae	*Solter hardei* Hölzel, 1968	
Myrmeleontidae	*Solter pallidus* Hölzel, 1982	
Myrmeleontidae	*Solter parvulus* Hölzel, 1988	
Myrmeleontidae	*Solter propheticus* Hölzel, 1980	+
Myrmeleontidae	*Solter tenellus* Hölzel, 1988	
Myrmeleontidae	*Solter vartianae* Hölzel, 1967	
Myrmeleontidae	*Solter virgilii* Navás, 1931	
Myrmeleontidae	*Solter wittmeri* Hölzel, 1982	
Myrmeleontidae	*Stenares irroratus* Navás, 1912	+
Myrmeleontidae	*Subgulina lineata* (Navás, 1913)	
Myrmeleontidae	*Syngenes arabicus* Kimmins, 1943	
Myrmeleontidae	*Tomatarella markli* Kimmins, 1952	
Nemopteridae	*Afghanocroce vartianorum* Hölzel, 1968	
Nemopteridae	*Croce aristata* (Klug, 1838)	+
Nemopteridae	*Croce schmidti* (Navas, 1927)	
Nemopteridae	*Dielocroce baudii* (Griffini, 1895)	
Nemopteridae	*Dielocroce berlandi* (Navás, 1936)	+
Nemopteridae	*Dielocroce chobauti* (McLachlan, 1898)	+
Nemopteridae	*Dielocroce elegans* (Alexandrov-Martynov, 1930)	+
Nemopteridae	*Dielocroce necrosia* (Navás, 1913)	
Nemopteridae	*Halter halteratus* (Forskål, 1775)	+
Nemopteridae	*Halter nutans* Navás, 1910	
Nemopteridae	*Necrophylus arenarius* Roux, 1833	
Sisyridae	*Sisyra nigra* (Retzius, 1783)	
Sisyridae	*Sisyra nilotica* Tjeder, 1957	

Key: + species is recorded in the current study.
